# Synthesis and antibacterial potential of novel thymol derivatives against methicillin-resistant *Staphylococcus aureus* and *P. aeruginosa* pathogenic bacteria

**DOI:** 10.3389/fchem.2024.1482852

**Published:** 2024-10-16

**Authors:** Ashutosh Shahi, Rakshit Manhas, Srija Bhattacharya, Arti Rathore, Puneet Kumar, Jayanta Samanta, Manish Kumar Sharma, Avisek Mahapa, Prasoon Gupta, Jasha Momo H. Anal

**Affiliations:** ^1^ Natural Products and Medicinal Chemistry Division, CSIR-Indian Institute of Integrative Medicine, (IIIM), Jammu, India; ^2^ Infectious Diseases Division, CSIR–IIIM, Jammu, India; ^3^ Academy of Scientific and Innovative Research (AcSIR), Ghaziabad, India; ^4^ Department of Chemistry, SRM Institute of Science and Technology, Kattankulathur, Tamil Nadu, India

**Keywords:** antibacterial, thymol derivatives, synergistic effect, antibiotic resistance, drug discovery

## Abstract

The increasing threat of antibiotic resistance has created an urgent need for new antibacterial agents, particularly plant-based natural compounds and their derivatives. Thymol, a natural monoterpenoid phenolic compound derived from *Monarda citriodora*, is known for its aromatic and therapeutic properties, including antibacterial activity. This study focuses on synthesizing dihydropyrimidinone and dihydropyridine derivatives of thymol and exploring their antibacterial properties. The synthesized compounds were tested for their *in vitro* antibacterial potential against pathogenic microorganisms, specifically *Pseudomonas aeruginosa* (Gram-negative) and methicillin-resistant *Staphylococcus aureus* (MRSA) (Gram-positive). Among the synthesized derivatives, compound 3i (ethyl 4-(4-hydroxy-5-isopropyl-2-methylphenyl)-2-imino-6-methyl-1,2,3,4-tetrahydropyrimidine-5-carboxylate) exhibited the most promising antibacterial activity, with minimum inhibitory concentration (MIC) values of 12.5 µM against *P. aeruginosa* and 50.0 µM against MRSA. Additionally, compound 3i demonstrated a synergistic effect when combined with vancomycin, enhancing its antibacterial efficacy. The optimum fractional inhibitory concentration index (FICI) observed was 0.10 and 0.5 for MRSA and *P. aeruginosa*, respectively, in combination with vancomycin. *In silico* analysis of the physiochemical properties of 3i indicated compliance with all drug-likeness rules. Furthermore, molecular docking studies revealed that compound 3i has a stronger binding affinity to the target protein than thymol, providing valuable insights into its potential mechanism of action.

## 1 Introduction

**FIGURE 1:**
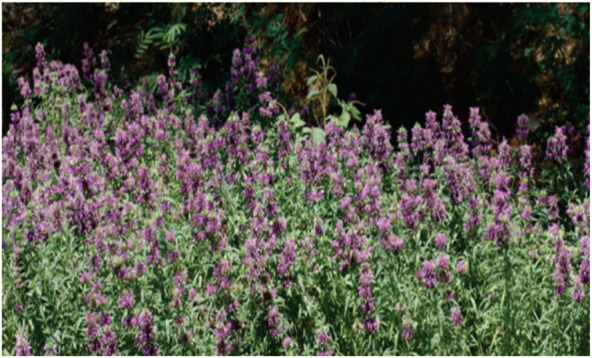
Jammu Monarda IIIM(J)MC-02.

Historically, natural products have played a crucial role in identifying and developing antibacterial agents. They have the potential to re-emerge as critical starting points in antibacterial discovery due to the emergence of antimicrobial resistance (AMR) in antibiotics (Moloney, 2016). Over the years, numerous studies have been extensively conducted on the antibacterial activity of natural sources, with a growing focus on plants, particularly herbs and spices (Abdou et al., 2007). Nowadays, more than 30,000 antibacterial compounds have been isolated from plants, and more than 1,340 plants have been found to exhibit specific antibacterial properties (Tajkarimi et al., 2010). The phytochemical compounds in these plants contain chemical functions or belong to families such as terpenes, isoflavonoids, aldehydes, ketones, and acids, which are critical constituents that exhibit antibacterial activity (Patra, 2006). Natural antibacterials can be used alone or in combinations as adjuvants in other applications like food preservation (Tiwari et al., 2009). *Monarda citriodora*, commonly known as Jammu Monarda, is a temperature-dependent plant. It is cultivated mainly in Jammu and Kashmir, Himachal Pradesh, Uttaranchal, and the higher lands of northeastern states (Figure 1). Recently, this plant has attracted the attention of research groups across the globe not only for its aromatic value but also for its potential as an antibacterial, antiviral, antifungal, antileishmanial, antitubercular, antioxidant, antiparasitic, and anticancer agent, as well as its use as kinase inhibitors and in few more drug applications all over the world (Mattarelli et al., 2017; Robledo et al., 2005; Sahoo et al., 2021). The essential oils collected from Monarda species have been analyzed and found to contain compounds like thymol, carvacrol, p-cymene, and their derivatives (Lawson et al., 2021). In the Lamiaceae family, thymol (2-isopropyl-5-methylphenol) is the main monoterpene phenol extracted from plants.

Multidrug-resistant bacterial strains have recently become a significant cause of persistent infections worldwide. This AMR has made effective drugs ineffective, making the bacterial infections untreatable. The emergence of AMR in bacterial pathogens has created an urgent need for novel antibacterial agents or alternative therapeutics (Ranjbar and Alam, 2024). According to a 2019 study, approximately 4.95 million deaths are associated with bacterial AMR, with AMR directly responsible for 1.27 million of these deaths (Murray et al., 2022). The ESKAPE group of pathogens (*Enterococcus faecium*, *Staphylococcus aureus*, *Klebsiella pneumoniae*, *Acinetobacter baumannii*, *P. aeruginosa*, and *Enterobacter* species) poses a global threat due to their concerningly rapid development of resistant properties. These pathogens are responsible for different deadly infections, eventually leading to death if left untreated (Ács et al., 2016). An earlier report showed the sensitivity of extracted essential oil containing thymol moiety from Monarda species against bacterial pathogens like *Pseudomonas aeruginosa* and methicillin-resistant *Staphylococcus aureus *(MRSA) (Utchariyakiat et al., 2016; Sahu and Siddiqui, 2016). In the present study, we have included *P. aeruginosa* (Gram-negative) and MRSA (Gram-positive bacteria, which are recognized as WHO priority pathogens. The development of novel therapies or adjuvants is urgently needed to combat resistant pathogens.

In this study, we isolated thymol, introduced an aldehyde group at the electron-rich *p*-position, and further derivatized thymol aldehyde to create a series of thymol dihydropyridine and dihydropyrimidinone derivatives. We synthesized nine thymol derivatives based on numerous potentials, which have antibacterial activities against Gram-negative and Gram-positive pathogens like *P. aeruginosa* and MRSA. These compounds have multiple implications in the pharmaceutical industry, and their derivatives possess different bioactivities. We further analyzed the synergistic potential of these derivatives using a checkerboard assay. This study explores the effectiveness of the newly synthesized thymol derivatives for their potential antibacterial properties that advance our battle against antibacterial resistance.

## 2 Materials and methods

### 2.1 Extraction and isolation

For isolation studies, 1.0 kg of dried marc of *Jammu Monarda* was collected from IIIM Farm (Chatha Farm) and was subjected to further extraction by ethyl acetate and methanol using a percolator. First, ethyl acetate was used for extraction. After filtration, ethyl acetate was evaporated completely using a rotary evaporator (Buchi R-200), and 55 gm of ethyl acetate extract was obtained. Similarly, after ethyl acetate, methanol was used for extraction, and after filtration, methanol was evaporated completely, and the MeOH extract (30.0 gm) was obtained. Upon further fractionation of the ethyl acetate fraction, six compounds were isolated, which were characterized as thymol, geraniol, limonene, carvacrol, cymene, and myrcene by using various spectroscopic techniques like NMR and mass spectroscopy. Thymol was found in the major quantity and all others in the minor quantity.

### 2.2 Preparation of 4-hydroxy-5-isopropyl-2-methylbenzaldehyde (thymol aldehyde)

**SCHEME 1:**
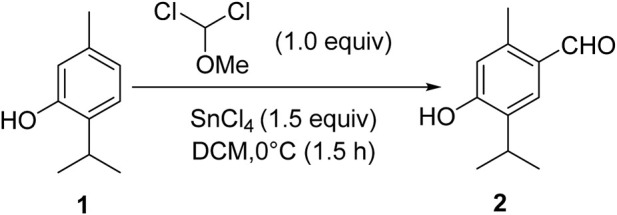
Synthesis of 4-hydroxy-5-isopropyl-2-methylbenzaldehyde (thymol aldehyde).

Thymol (1.0 mmol), isolated from the marc of *Jammu Monarda*, and dichloromethyl methyl ether (1.0 mmol) in dichloromethane (25 mL) were mixed in a round bottom flask and stirred at 0°C for the initial half an hour. Then, tin chloride (1.5 mmol) was added dropwise to the reaction mixture, and stirring was carefully continued at room temperature for 3 h. After the completion of the reaction (monitored by TLC) to ascertain product formation, it was quenched slowly with ice water. The product was extracted using ethyl acetate. The combined organic layer was dried with anhydrous sodium sulfate and concentrated in rota-vapor, and the residue was subjected to silica gel column chromatography using hexane: ethyl acetate to afford the pure product, thymol aldehyde, 2 (Scheme 1).

**FIGURE 2:**
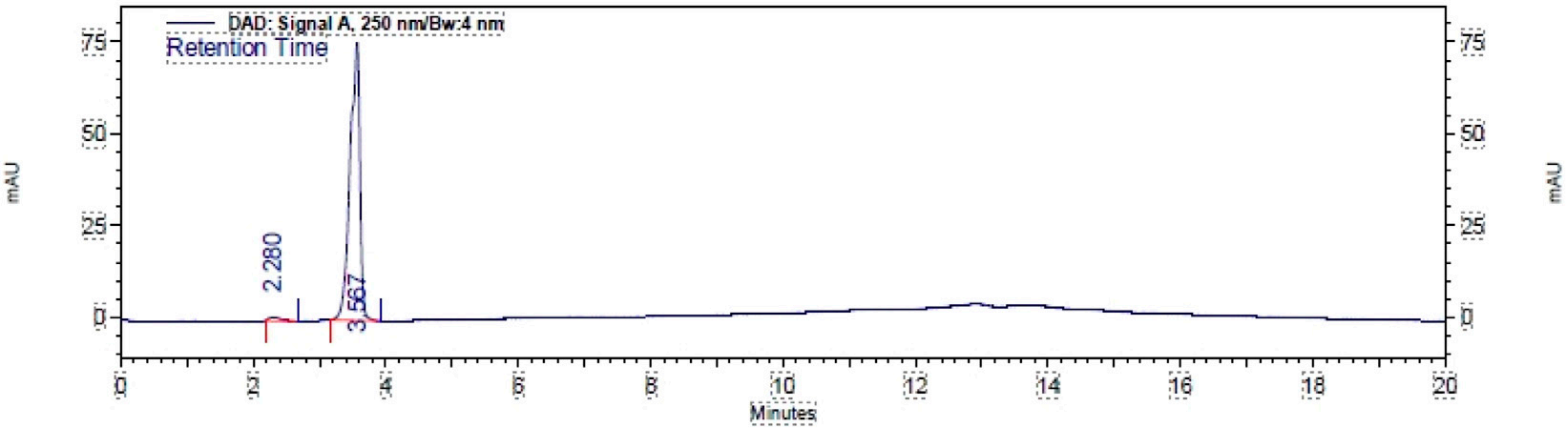
HPLC chromatogram of thymol aldehyde (2).

High-performance liquid chromatography (HPLC) detected 98% of Figure 2, as shown in the chromatogram. HPLC was run using a gradient method to ensure the detection of any other products that may have formed on the C_18_ column. The mobile phase consisted of acetonitrile (MeCN) and water, with eluting conditions maintained throughout a run time of 20 min at a flow rate of 1.0 mL/min. The eluting solvent mixture MeCN/H_2_O composition started at 70:30, then changed to 80:20 and 90:10, and returned to 80:20 and 70:30, with an interval of 5.0 min each, over a run time of 20.0 min. The retention time, t_R_, of compound 2 was detected at 3.567 with an area percentage of 98.445.

### 2.3 General procedure for the synthesis of thymol derivatives

For the preparation of compounds 3(a–c), the prepared thymol aldehyde (1.0 mmol) and β-keto ester (2.0 mmol) in ethanol (25 mL) were placed in a round bottom flask. Then, ammonia (2.0 mmol) was added dropwise, and the mixture was stirred for 5 h at room temperature. After the completion of the reaction (product monitored by TLC), it was slowly quenched with H_2_O. The product was extracted using ethyl acetate. The combined organic layer was dried with anhydrous sodium sulfate and concentrated in rota-vapor, and the residue was subjected to silica gel column chromatography using hexane: ethyl acetate to afford the pure product.

For compounds 3(d–h), thymol aldehyde (1.0 mmol), β-keto ester (2.0 mmol), and urea (1.5 mmol) or thio urea (1.5 mmol) in acetic acid (25 mL) were placed in a round bottom flask. Then, zinc chloride was added in a catalytic amount (0.3 mmol), and the mixture was reflexed at 80°C for 5 h After the completion of the reaction (product monitored by TLC), it was slowly quenched with sodium bicarbonate dissolved in water (for neutralizing acid), and the pure product was collected in the same manner as 1,4-dihydropyrimidine derivatives.

For the synthesis of compound 3i, thymol aldehyde 2 (1.0 mmol), β-keto ester (2.0 mmol), and guanidine (1.5 mmol) in ethanol (25 mL) were placed in the round bottom flask. Then, sodium carbonate was added in a catalytic amount (0.3 mmol), and the mixture was refluxed at 80°C for 5 h After the completion of the reaction (product monitored by TLC), the pure product was collected in the same manner as 1,4-dihydropyrimidinone derivatives.

### 2.4 Biology

#### 2.4.1 Bacterial strain and growth

various bacterial strains were used for this study. These included *P. aeruginosa* (Schroeter) Migula 27,853, a Gram-negative strain, and MRSA, a Gram-positive strain. Additionally, *Escherichia coli*, *K. pneumoniae*, and *Mycobacterium smegmatis* were also included. The ESKAPE pathogens were grown in LB media, and MB7H9 media (supplemented with 2% glycerol) was used for mycobacterial growth. All the strains were stored at −80°C (20% glycerol stock). A 20 µL sample of bacteria from the glycerol stock was added to 10 mL of LB/MB7H9 media to test their antibacterial properties. The cultures were then maintained at 37°C overnight with continuous shaking.

#### 2.4.2 Minimum inhibitory concentration (MIC)

The thymol derivatives 3(a–i) were evaluated for their *in vitro* antibacterial potential using a 96-well microtiter plate method with some modifications according to the CLSI guidelines (Wang et al., 2021). Before the assay, the thymol derivatives 3(a–i) were dissolved in DMSO to prepare their (5 mg/mL) sample stocks, and reference drug vancomycin was dissolved in distilled water to prepare a stock of 1.0 mg/mL. The indicator solution of resazurin (indicator) was prepared as 0.04% in PBS. First, bacterial strains were grown overnight in LB broth at 37°C in a shaker incubator. In addition, 100 μL of LB media was added to a 96-well flat-bottom microtiter plate the next day. Two-fold concentrations of thymol derivatives were added horizontally in row 1, starting from well 1 to well 12. The compounds were then serially diluted vertically for each row up to the eighth well. Finally, 100.0 µL bacterial cultures at final OD_600_∼0.05 were added to each well. The plates were sealed with parafilm and incubated at 37 °C for 24 h. After incubation, 10 µL of the resazurin solution was added to each well, and the plates were incubated at 37 °C for 1 h. The optical density (OD) was observed at 570 nm on the microplate reader (TECAN Infinity 200 pro). The MIC is estimated as the minimum concentration of the compounds at which the resazurin’s color did not reduce to pink. The experiment was performed in three biological replicates against all the pathogens.

#### 2.4.3 Minimum bactericidal concentration (MBC)

MBC was considered the lowest compound concentration, where no visible colonies were observed. To perform the MBC assay, a total volume of 10 µL culture was removed from the wells of microtiter MIC plates (treated with compound 3i) and plated on LB agar. The plates were kept for 24-h incubation at 37°C (Rodríguez-Melcón et al., 2021). The MBC value was obtained from the plates, where no visible growth was observed at a particular concentration.

#### 2.4.4 Synergistic activity

The synergistic study was performed between thymol derivatives and reference drug vancomycin against *P. aeruginosa* and MRSA, according to the National Committee for Clinical Laboratory Standards (NCCLS) guidelines (Utchariyakiat et al., 2016). In brief, a two-fold concentration of each drug combination and stock solution was prepared before testing (Orhan et al., 2005). Bacterial cultures were maintained in an LB medium overnight. The next day, one hundred microliters of LB media were added to 96-well microtiter plates, where compound 3i (vertically) and vancomycin (horizontally) were added and serially diluted. One hundred microliters (100 µL) of bacterial suspension (at OD 0.05) was added to the plates and incubated at 37°C for 24 h. To find the synergy, the fractional inhibitory concentration index (FICI) was calculated for each drug combination in a checkerboard assay. The FICI was calculated using the following formula:

$$\text{FICI}=\text{FICA}+\text{FICB}=\left(\frac{\text{CA}}{\text{MICA}}\right)+\left(\frac{\text{CB}}{\text{MICB}}\right),$$

where CA and CB are the MICs of drugs A and B in combination (in a single well) and MICA and MICB are the MICs of each drug individually. The following interpretation criteria were followed for the checkerboard assay: the FICI value ≤0.5 indicated synergy, the FICI value between 0.5 and 4 indicated indifference or additive, and FICI >4 indicated antagonism.

## 3 Results

Thymol aldehyde (2) was synthesized from thymol, which is isolated from *Jammu Monarda* (Scheme 1) by the Rieche formylation, which is an important method for synthesizing aromatic aldehydes. In this process, dichloromethyl methyl ether acts as a formylation agent for electron-rich aromatic compounds in the presence of a Lewis acid such as titanium tetrachloride Polat and Cakici, 2022). In this reaction, we have used tin (iv) tetrachloride. This formylation method of converting thymol to its aldehyde allows further transformations to an extensive possibility for essential pharmaceutical ingredients.

**SCHEME 2:**
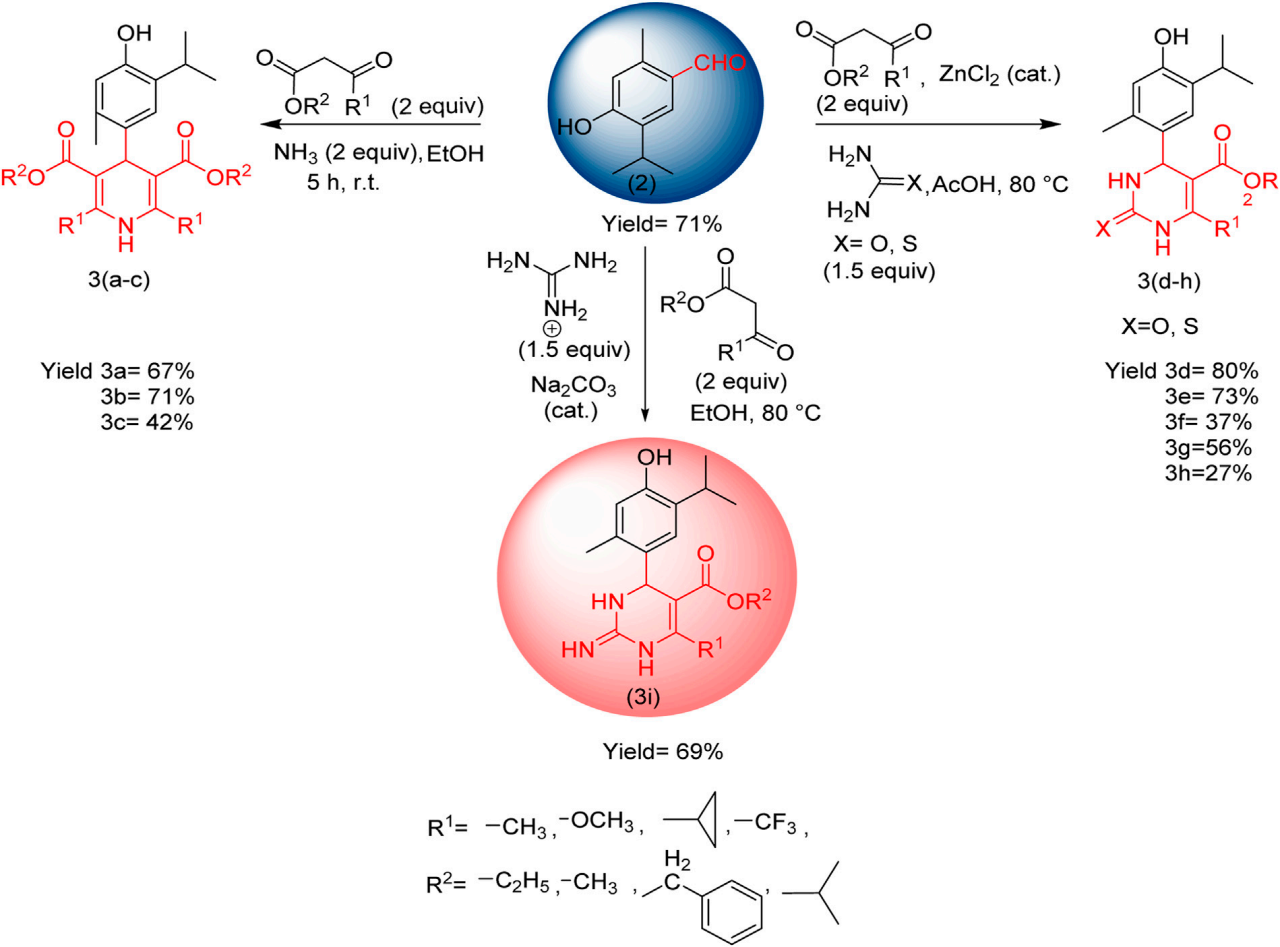
Synthesis of thymol derivatives.

Furthermore, a modification of thymol aldehyde and synthesis of compounds of pyridine and pyrimidinone derivatives are described (Scheme 2).

Compounds 3(a–c) were synthesized via the Hantzsch dihydropyridine (DPH) method using thymol aldehyde, β-keto ester (Supplementary Figure S1), and ammonia, which was added dropwise at room temperature in ethanol, and compounds 3(d–h) were synthesized using urea or thiourea instead of ammonia in acetic acid, with a catalytic amount of zinc chloride, and the mixture was refluxed at 80 °C for 5 h. In the case of compound 3i, guanidine was added in ethanol, with sodium carbonate acting as a catalyst, and the mixture at was refluxed 80 °C for 5 h (Tsvetkov et al., 2024).

A multicomponent reaction (MCR) is a synthetic methodology in which three or more reactants combine in a single vessel to form a new product. The defining aspect of MCRs is that the final products contain nearly all substrate portions, generating minimal by-products, which are helpful for drug discovery activities. MCR is a highly ideal and eco-friendly reaction system. Target compounds can be obtained in one pot with fewer steps (Weber, 2002). Thymol aldehyde converted from thymol is a highly electron-rich center susceptible to electrophilic aromatic substitution using the Rieche formylation method. Rieche formylation gives excellent yields and regiospecificity. It does not lead to further formylation (Garcıa and Nicolás, 2003; Alan et al., 2010; Jun and Yinjuan, 2002; Oliver, 2000; Majellaro et al., 2020).

**FIGURE 3:**
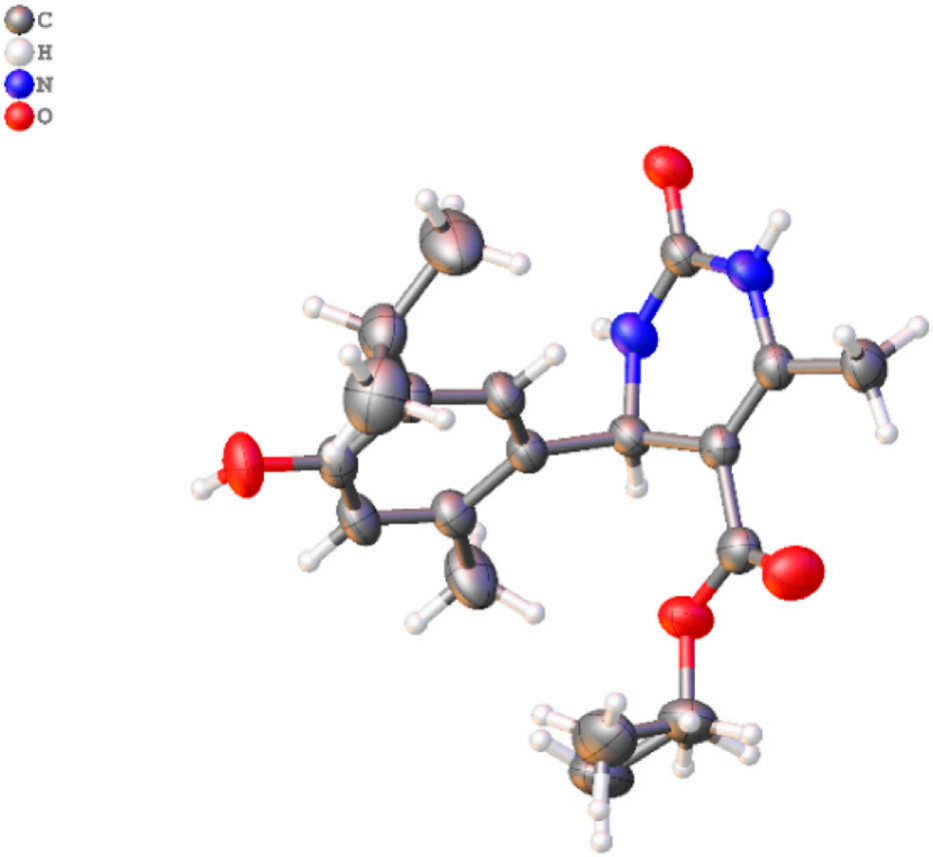
Molecular structure of 3d. The disordered part is removed for clarity. Thermal ellipsoids are shown at a 50% probability level (color code: C = gray, H = white, N = blue, and O = red) (CCDC2287405).

The stereochemistry of the synthesized compounds 3 (a–c) and 3 (d–i) can theoretically determine whether they exist as racemic mixtures or exhibit enantiomeric excess. We optimized the 3D geometries of the synthesized molecules 3(d–i) using the DFT/B3LYP method and 6-31G (d,p) basis set. Our theoretical calculations indicate that in the case of compounds 3(d–g) (Table 1; Figure 3), the S-configuration exhibits lower energy and a decreased dipole moment compared to the R-configuration. This difference is attributed to a carbonyl group (C=O) within the dihydropyrimidine ring containing an electronegative oxygen atom. It confirms that the S-isomer is theoretically more stable and predominant compared to the R-isomer. Conversely, for compounds 3h and 3i (Table 1; Supplementary Figure S3), the R-isomer displays lower energy and dipole moment due to C=S and C=NH groups within the dihydropyrimidine ring, respectively. The reduced electronegativity of sulfur and nitrogen atoms compared to oxygen theoretically contributes to the stability and predominant presence of the R-isomer in these compounds.

**TABLE 1:** Energies and dipole moments of the compounds were calculated using the DFT/B3LYP method and 6-31G (d,*p*) basis set.

Compound	*R-configuration*		*S-configuration*	
	Energy (*Hartree*)	Dipole moment (*Debye*)	Energy (*Hartree*)	Dipole moment (*Debye*)
3 days	−1,110.852098	3.782651	−1,110.852146 (*min*)	3.674134
3e	−1,302.589632	3.840660	−1,302.589682 (*min*)	3.774777
3f	−1,447.871466	4.457909	−1,447.877407 (*min*)	3.765771
3g	−1,148.914783	3.840445	−1,148.914825 (*min*)	3.740330
3h	−1,433.814023 (*min*)	4.028652	−1,433.813994	4.100613
3i	−1,090.961189 (*min*)	1.677703	−1,090.961175	1.860417

### 3.1 Crystal study

A single X-ray diffraction technique validated the molecular structure of compound 3d. The crystal was grown in a diethyl ether: MeOH solvent mixture by slow evaporation at room temperature. The details of the crystal and data refinement are given in Supplementary Material, whereas the molecular structure and crystal (Figure 3) were determined further to verify the structure of the formation of this pharmacophore.

### 3.2 Antibacterial properties of thymol derivatives

The thymol derivatives were evaluated for their antibacterial efficacies against pathogens like *P. aeruginosa* and MRSA. We conducted MIC assays on the thymol derivatives, specifically compounds 3(a–i), to assess their antibacterial activity. Following the guidelines of the Clinical and Laboratory Standards Institute (CLSI) with some modifications (Humphries et al., 2018), we performed a microdilution assay and obtained MIC values for these compounds against different pathogens. The reference antibacterial drug, vancomycin, was also included in the assay. Compound 3i exhibited significant antibacterial activity against *P. aeruginosa* and MRSA among all the synthesized thymol derivatives. Compound 3i inhibited the growth of *P. aeruginosa* and MRSA with MIC values ranging from 12.5 to 50.0 µM, respectively. The remaining derivatives also demonstrated antibacterial efficacy; however, their antibacterial activity was observed at higher concentrations (>100 µM) against broad-spectrum pathogens such as *E. coli*, *K. pneumoniae*, and *M. smegmatis* (Supplementary Table S1).

We have also included standard positive control vancomycin in the study, with MIC values of 6.25 and 3.1 μM against *P. aeruginosa* and MRSA, respectively.

**FIGURE 4:**
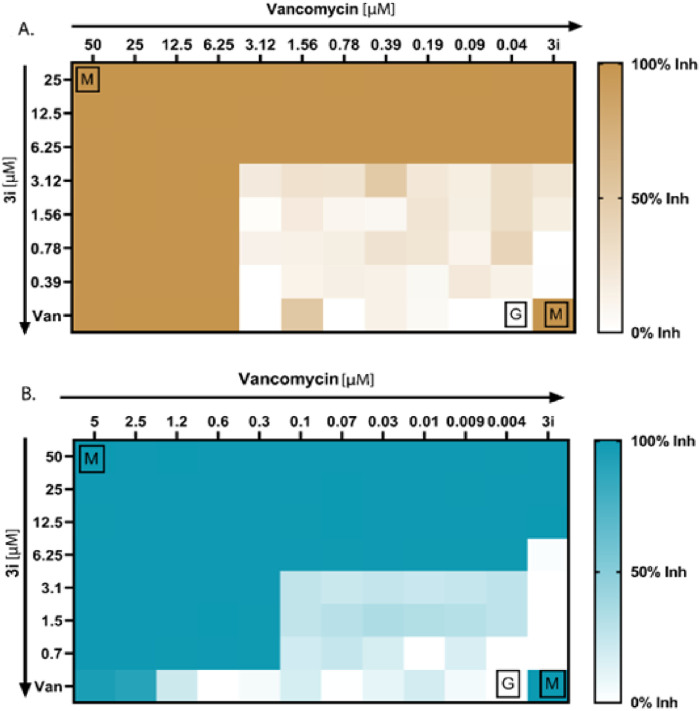
Antibacterial synergy of compound 3i. **(A)** Antibacterial synergy of compound 3i with vancomycin against *P. aeruginosa*. **(B)** Antibacterial synergy of compound 3i with vancomycin against MRSA.

Next, we determined the bactericidal properties of the thymol derivative (3i). We performed MBC experiments. Figure 4 illustrates the MBC values of compound 3i. As depicted in the agar plate, no visible growth of MRSA was observed at concentrations of 50.0 and 100.0 µM, while bacterial colonies were observed at 25 µM. This suggests that compound 3i exhibits a bactericidal effect at 50 µM against MRSA. Additionally, no colonies of *Pseudomonas* were detected at concentrations of 12.5 µM or higher, indicating the potent bactericidal activity of compound 3i against *Pseudomonas* at the same concentration. The antibacterial activity of thymol and its derivative 3i against *P. aeruginosa* (Gram-negative) and MRSA (methicillin-resistant *S. aureus*) (Gram-positive) bacterial strains, with vancomycin used as the standard (Table 2), indicated good activity against clinically isolated bacterial strains.

**TABLE 2:** MIC values of compounds in µM against clinically isolated bacterial strains.

Compound	*Pseudomonas aeruginosa*	Methicillin-resistant *Staphylococcus aureus*
1	>100	>100
3i	12.5	50
Vancomycin	6.25	3.1

### 3.3 Compound 3i synergizes vancomycin activity against MRSA and *P. aeruginosa*

We conducted checkerboard assays to determine whether the thymol derivative 3i and the reference drug vancomycin work synergistically against *P. aeruginosa* and MRSA. Combining drugs can synergistically reduce the need for higher doses of antibiotics and minimize adverse effects. We tested 77 possible combinations, as shown in Figures 4A, B, to determine the synergistic combinations among the thymol derivative 3i with vancomycin. We calculated the FICI using the standard formula specified in the method section to assess the number of synergies, additives, or antagonisms. Our result showed that for *P. aeruginosa*, four combinations of 3i and vancomycin were synergistic, with FICI values below 0.5. We also observed 36 no interactions with FICI values above 0.5 and up to 4, and 9 antagonistic combinations between the two drugs have been observed. Additionally, the checkerboard assay between 3i and vancomycin against MRSA yielded 32 possible synergistic combinations with FICI values below or equal to 0.5, indicating a synergy effect. We also identified 27 no interactions with FICI values above 0.5 and up to 4. Notably, no antagonism exists, as indicated by FICI values above 4. These findings suggest that combining 3i with vancomycin can enhance antibacterial activity. The synergistic combinations identified in this assay could explore the possibility of reducing drug doses and minimizing associated side effects while maintaining effective antibacterial activity against *P. aeruginosa* and MRSA.

The present study demonstrated the synthesis of novel thymol derivatives. It investigated the antibacterial potential of those thymol derivatives, focusing on bioactive compound 3i, against a panel of bacterial pathogens. Thymol is a natural compound with antibacterial properties, reported earlier in numerous studies (Zhang et al., 2021). Our MIC assays with the thymol derivatives revealed that compound 3i showed significant antibacterial activity among the tested molecules against *P. aeruginosa* and MRSA, WHO-priority pathogens. Significantly, compound 3i displayed lower MIC values of 12.5 µM/1.9 μg/mL and 50 µM/7.5 μg/mL against MRSA and *P. aeruginosa*, respectively, indicating improved potency compared to thymol and its derivatives 3(a-i). The MIC and MBC values of the native thymol ranged from 250 μg/mL to 1,000 μg/mL, respectively, against *S. aureus*, whereas for *P. aeruginosa*, they were greater than 1,000 μg/mL (Gan et al., 2023; Kim et al., 2022). This suggests that specific modifications in the chemical structure, as seen in compound 3i, can lead to heightened antibacterial efficacy against the examined pathogens. Additionally, our MBC experiments further supported the strong bactericidal effects of compound 3i at lower concentrations (50.0 µM against MRSA and 12.5 µM against *Pseudomonas*). The effective bactericidal activity at such low concentrations is a notable advantage and indicates compound 3i’s potential as an antibacterial compound. Another significant feature of this study was investigating the synergy between compound 3i and the reference antibacterial, vancomycin. It is used to treat MRSA infections. The usefulness of drug combinations has become an essential strategy to combat antibacterial resistance and improve treatment outcomes (Liang et al., 2007). The checkerboard assay allowed us to search a wide range of combinations (77 combinations) and determine suitable antibacterial synergy. Our checkerboard assay revealed that for *P. aeruginosa*, four combinations of compound 3i and vancomycin were synergistic (FICI values below 0.5). Similarly, for MRSA, 32 synergistic combinations were observed. The relative analysis between the natural product thymol and compound 3i reveals its antibacterial potency, especially against *P. aeruginosa* and MRSA (Table 2). Additionally, the synergistic effect observed when combining compound 3i with vancomycin highlights its potential in combination therapies. Our findings emphasize the significance of chemical modifications in enhancing the antibacterial properties of natural compounds and their potential contribution to combating antibacterial resistance.

### 3.4 Molecular docking analysis and *in silico* physicochemical studies

**FIGURE 5:**
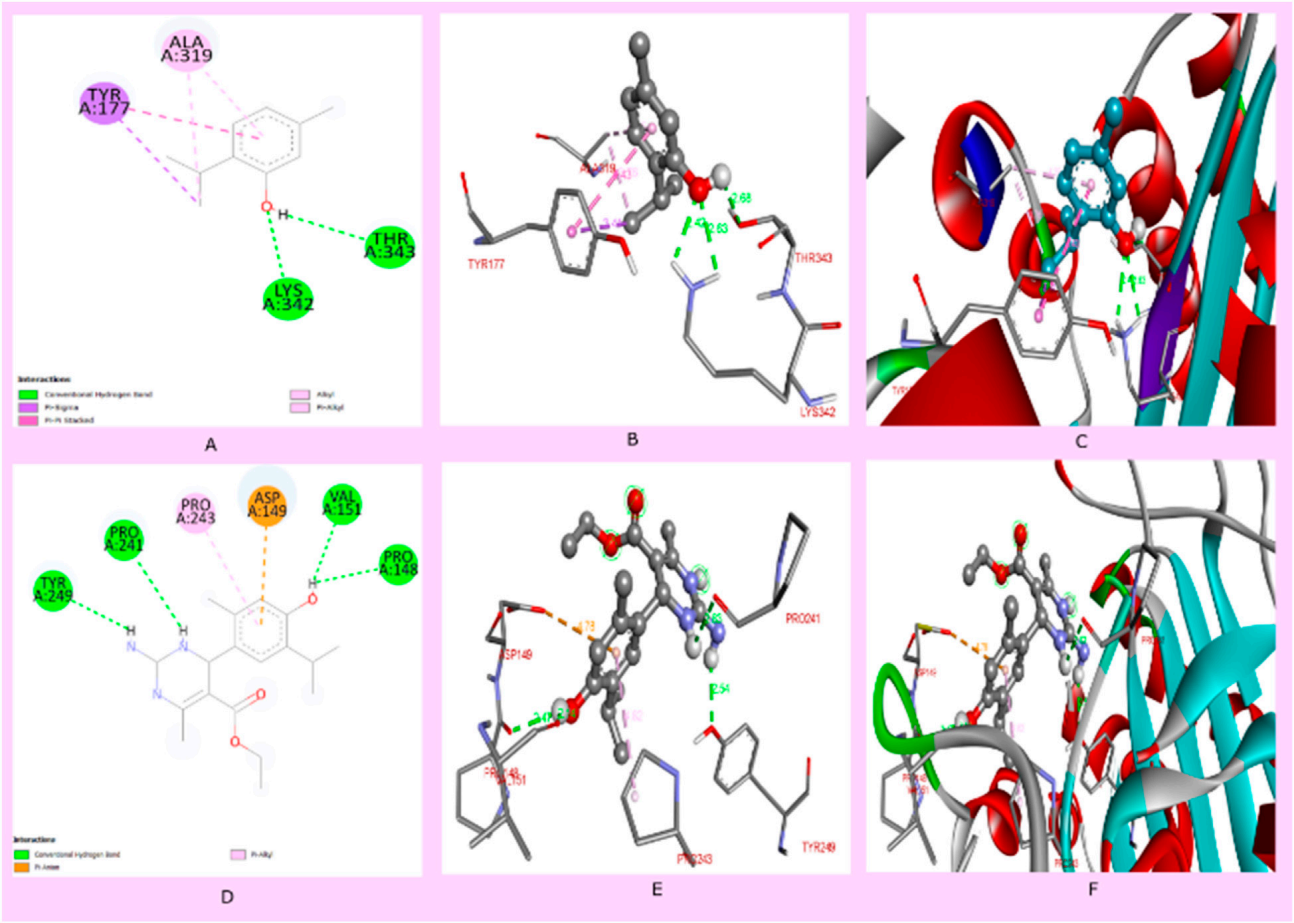
**(A)** Two-dimensional interaction of thymol with proteins (pdb id: 4HEF), (B) 3D interaction of thymol with amino acids of proteins, **(C)** 3D interaction of thymol with a protein receptor, **(D)** 2D interaction of compound 3i with proteins (PDB ID: 4HEF), **(E)** 3D interaction of compound 3i with amino acids of proteins, and **(F)** 3D interaction of thymol with a protein receptor.

In the current drug development approaches, computational processes are utilized to predict potentially effective drug similarity molecules to direct or avoid synthesizing more active compounds. Physicochemical properties play an essential role during the development of drugs. The prediction of parameters using SwissADME to evaluate pharmacokinetics, drug-likeness, and medicinal chemistry supports the *in vitro* analysis of compounds, which exhibited that compound 3i displayed more drug-like properties than thymol. Compound 3i obeys all drug-likeness rules, including Lipinski’s rule, Ghose’s rule, Veber’s rule, Egan’s rule, and Muegge’s rule, while thymol violates Ghose’s rule and Muegge’s rule of drug-likeness. Moreover, the Lipinski rule of five (RO5), based on molecular properties, molecular weight, numbers of hydrogen acceptors (H-ba), number of hydrogen donors (H-bd), and LogP values, was widely used in the selection criterion for an active drug molecule (Table 3). Compound 3i has a better binding affinity (docking score −6.9 kcal/mol), whereas thymol 1 shows a binding affinity (docking score −5.2 kcal/mol) to the protein interaction. Thymol interacted with amino acids LYS342 and, THR343 by conventional hydrogen bond, TRY177 by pi-sigma bond, and ALA319 by pi–pi stacking interaction. It is evident that docking compound 3i interacts with the amino acids of proteins, including TYR249, PRO241, VAL151, and PRO148 by conventional hydrogen bond, PRO243 by pi-alkyl bonding, and ASP149 by pi-sigma bonding. Compound 3i displayed better hydrogen bonding interaction than the parent compound (thymol) with the protein (Figure 5).

**TABLE 3:** Prediction of physicochemical properties of parent thymol and compound 3i.

Parameter	Thymol	3i
Molecular formula	C_10_H_14_O	C_18_H_25_N_3_O_3_
Molecular weight (g/mol)	150.100	331.190
H-bond acceptors	1	4
H-bond donors	1	4
TPSA (Å^2^)	20.23	94.44
Log Po/w (iLOGP)	2.32	2.59
Solubility (mg/mL)	9.74e^−02^	1.36e^−01^
Drug-likeness Lipinski (violation)	Yes (0)	Yes (0)
Drug-likeness Ghose	No (1)	Yes
Drug-likeness Veber	Yes	Yes
Drug-likeness Egan	Yes	Yes
Drug-likeness Muegge (violation)	No (2)	Yes (0)
Lead-likeness	No	Yes
Bioavailability score	0.55	0.55
GI absorption	High	High
BBB permeation	Yes	No

## 4 Discussion

Thymol exhibits significant antibacterial properties, inhibiting the growth of Gram-positive and Gram-negative bacteria (Palaniappan et al., 2010). The hydroxyl group in thymol and the LogP ratio of thymol, measured at 3.37, also contribute significantly to the antibacterial effect (Koroch et al., 2007; Anna et al., 2016). According to previous studies, the presence of the 1,4-dihydropyridine (1,4-DHP) central core is essential for antibacterial activity (Malhi et al., 2022). Investigations into dihydropyrimidine derivatives indicate that compounds containing thio- and oxo-groups possess enhanced potency (Yadlapalli et al., 2012). Thymol, pyridine derivatives, and pyrimidine derivatives each have individual antibacterial activity, suggesting the potential for enhanced activity by combining them.

**FIGURE 6:**
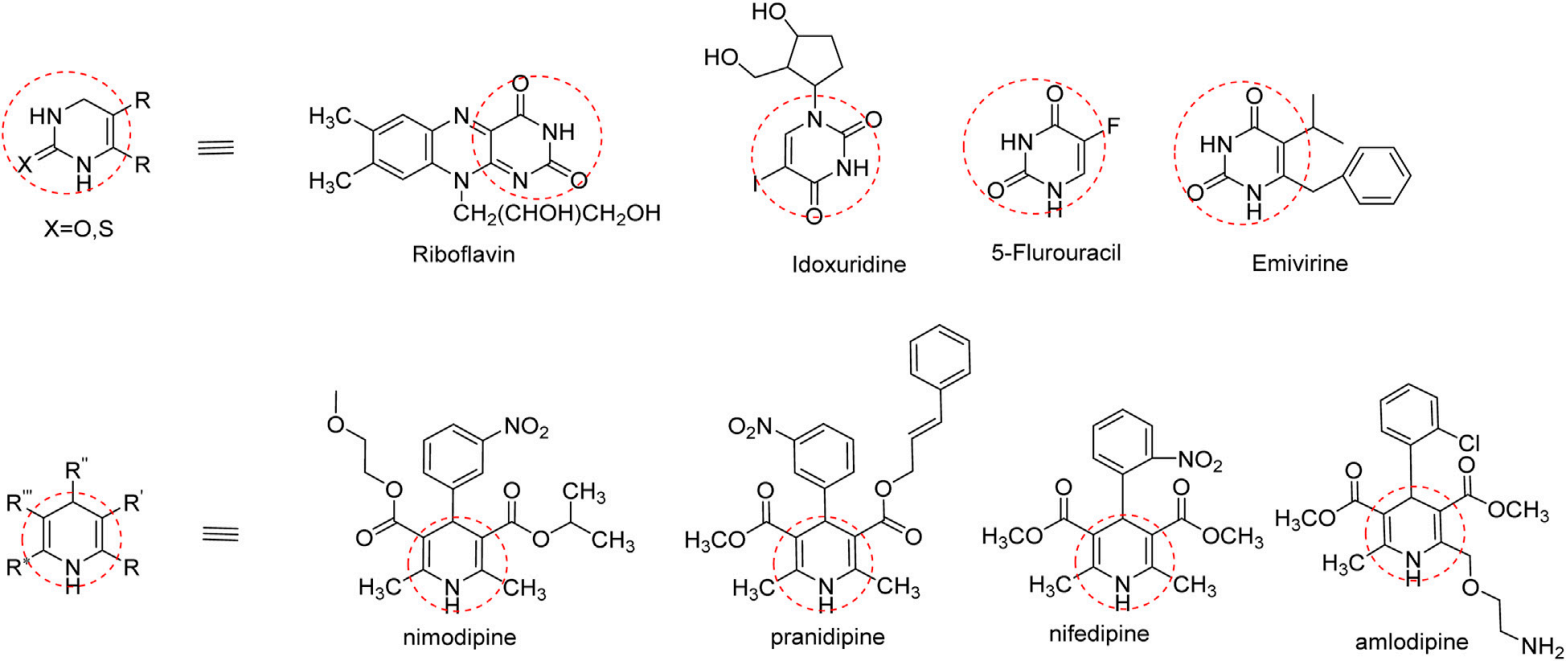
Dihydropyridine and dihydropyrimidinone pharmacophore-containing drugs.

In addition to natural thymol, the antibacterial efficacies of the derivatives were reported against different pathogens; the aryl-azo-thymol derivatives, which were synthesized, showed promising activity against MRSA, with an MIC value of 40 μg/mL. Another thymol oxypropanolamine compound (1-([cyclohex-1-en-1-ylmethyl] amino)-3-(2-isopropyl-5-methylphenoxy) propan-2-ol) exhibited efficient activity against *A. baumannii,* with a zone of inhibition of 3 mm. A halogenated thymol derivative, chloro-thymol (4-chloro-2-isopropyl-5-methylphenol), possesses significant activity at 12.5 and 25 μg/mL against *S. aureus* and *Staphylococcus epidermis*, respectively (Si et al., 2023).Therefore, we incorporated a nitrogen base into these pharmacophores to improve their activity. This base is essential for the nucleic acid biosynthesis pathway and is crucial for cell survival and various biological processes (Sienkiewicz et al., 2013). Interestingly, several FDA-approved drugs contain dihydropyridine, and dihydropyrimidine pharmacophores are presented in Figure 6 (Kaur et al., 2017).

## 5 Conclusion

Three dihydropyridine and six dihydropyrimidinone derivatives were obtained by converting thymol into its thymol aldehyde (Scheme 2). The antibacterial evaluation against various pathogenic bacteria led to a significant finding: among the nine compounds 3(a–i), compound 3i exhibited significant activity against *P. aeruginosa* and MRSA, with MIC values of 12.5 µM and 50.0 µM, respectively. Compound 3i shows broad-spectrum bactericidal activity against Gram-negative and Gram-positive pathogens and a synergistic effect for its combination therapy. The antibacterial characterization of compound 3i as a potent antibacterial agent offers valuable information for further studies and drug development. These findings indicate that these thymol derivatives have the potential to be novel antibacterial agents in combating microbial infections and contribute to the fight against antibiotic resistance by using biomass-derived waste from plant sources in a sustainable manner. Furthermore, the exploration of structural optimization for its better efficacy, toxicity, and safety will be carried out in future *in vivo* studies. We believe that such endeavors play a crucial role in strategizing the continuous battle against antibacterial resistance, emphasizing the significance of natural products as a reservoir for innovative drug discovery and development with potential pharmaceutical industrial applications.

## Data Availability

The datasets presented in this study can be found in online repositories. The names of the repository/repositories and accession number(s) can be found in the article/Supplementary Material.
