# Supplementary material for: Synthesis and antibacterial potential of novel thymol derivatives against methicillin-resistant Staphylococcus aureus and P. aeruginosa pathogenic bacteria
Source: Front Chem. 2024 Oct 16;12:1482852. doi: 10.3389/fchem.2024.1482852 (PMC11523063; doi:10.3389/fchem.2024.1482852)
Supplement: Supplementary file 1 [file DataSheet1.pdf]

Supporting Information

Synthesis and antibacterial potential of novel thymol derivatives against methicillin-resistant *Staphylococcus aureus* and *P. aeruginosa* pathogenic bacteria

Ashutosh Shahi,[#1] Rakshit Manhas,[#2] Srija Bhattacharya[1], Arti Rathore[2,3], Puneet Kumar[1,3], Jayanta Samanta[4], Manish Kumar Sharma[1], Avisek Mahapa,[2,3]* Prasoon Gupta,[1,3]* and Jasha Momo H. Anal [1,3]*

[1]Natural Products and Medicinal Chemistry Division, CSIR-Indian Institute of Integrative Medicine, Canal Road, Jammu-180001, India

[2]Infectious Diseases Division, CSIR–IIIM, Canal Road, Jammu-180001, India

[3]Academy of Scientific and Innovative Research (AcSIR), Ghaziabad-201002, India

[4]Department of Chemistry, SRM Institute of Science and Technology, Kattankulathur--603 203, Tamil Nadu, India.

# Contributed equally

Correspondence to: avisek.mahapa@iiim.res.in; guptap@iiim.ac.in; hmunshel.jasha@iiim.res.in.

Table of Contents

Contents Page No.	Contents Page No.

1. General Information…………………………………...................................	3
2. General Experimental Procedure...………………………………...................	4-5
3. Characterization Data of Compounds 1-3i..………………………..................	5-11
4. NMR Spectra……………………………………………………..................	11-22
5. Crystal Experiment………………………………………………....................	23
6. Theoretically Optimized Structures of the Molecules…………….....................	23-24
7. Biology Experimental Section: Antimicrobial and synergistic effect of thymol derivatives with currently used antibiotics (Vancomycin):..............	25-30
8. Referances	30-31

1. General Information

Materials: All glassware was oven-dried (90 °C). Unless mentioned, chemicals and solvents were purchased in high purity grade from commercial suppliers and used without further purification.

Chromatography: Thin layer chromatography (TLC) was carried out on Merck silica plates (60F- 254) and components were visualised by observation under UV light or by treating the plates with a mixture of ceric ammonium nitrate and sulfuric acid solution followed by heating. Silica gel chromatography was performed using silica gel (60–120 or 100-200 mesh) using hexane: ethyl acetate and DCM: MeOH as the eluent to obtain the pure products.

Characterization: NMR spectra for the characterization of compounds were recorded on Bruker Avance DPX FT-NMR 400 MHz instrument (^{1}H) at 400 MHz and (^{13}C) at 100 MHz respectively. Chemical shifts (δ) are reported in ppm, using the residual solvent peak in $CDCl_3$ (δH = 7.26 and δC = 77.16 ppm) and CD_3OD (δH = 4.80 and δc = 48.28ppm) as an internal reference and coupling constants (*J*) are given in hertz (Hz). The following abbreviations were used to explain the multiplicities: s = singlet, d = doublet, t = triplet, q = quartet, and m = multiplet. High-resolution mass Spectra (HRMS) were recorded using Waters XEVO-G2-XS-Q-TOF mass spectrometer using electron spray ionization. Reagents used were mostly purchased from Sigma Aldrich, TCI, Alfa Aesar and Avra Synthesis.

Experimental details: Unless mentioned, reactions were performed in an open atmosphere at room temperature (25-30 °C) in a 50ml round bottom flask. Reagents used were mostly purchased from Sigma Aldrich, TCI, Alfa Aesar and Avra Synthesis. The solvent used for the reaction purpose is of commercial grade and used without further purification.

2. General Experimental Procedure

For DPH thymol derivatives **3(a-c)** the ^{1}H NMR of the C-4 aryl chemical shift was observed at sharp singlet in the range from 6.0-6.7 ppm, for ^{13}C NMR C-4 aryl substituents at a range from 38.71-26.60 ppm, and C-3 and C-5 with ester linkage was observed at 105.11 ppm for **3a**, 60.63 ppm for **3b**, and 105.13 ppm for **3c.** The C-2 and C-6 attached to the nitrogen in the pyridine ring were observed at 151.09 for **3a**, 155.50 for **3b,** and 155.58 for **3c.** The carbonyl for the three DPHs was observed at 168.24, 168.17, and 168.55 ppm. Similarly, for DPHMs thymol derivatives **3(d-i),** the ^{1}H NMR of the C-4 aryl chemical shift was observed at sharp singlet in the range from 4.87-6.7 ppm, for ^{13}C NMR C-4 aryl substituents at a range from 50.30-60.01 ppm, and C-5 carbon with ester linkage was observed in the range of 101.10-107.19. The C-2 carbon where there was a carbonyl bond in the dihydropyrimidinone ring for **3d**, **3e**, **3f**, and **3g** was observed in a range from 150.66-155.44 ppm, whereas for **3h** at 173.66 ppm due to C-S double bond. The carbonyl ester for the DPHMs was observed in the range of 166.38-169.71 ppm, depending upon different substituents attached to the ring.

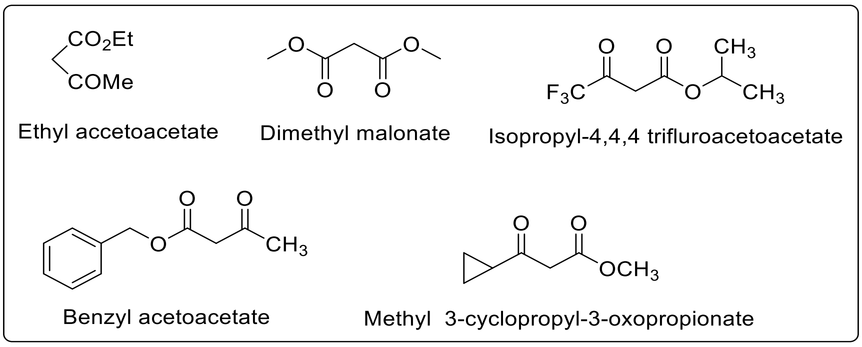

Figure 1: Substituted β-keto ester involves in this study

2.1 HPLC chromatogram of thymol aldehyde 2

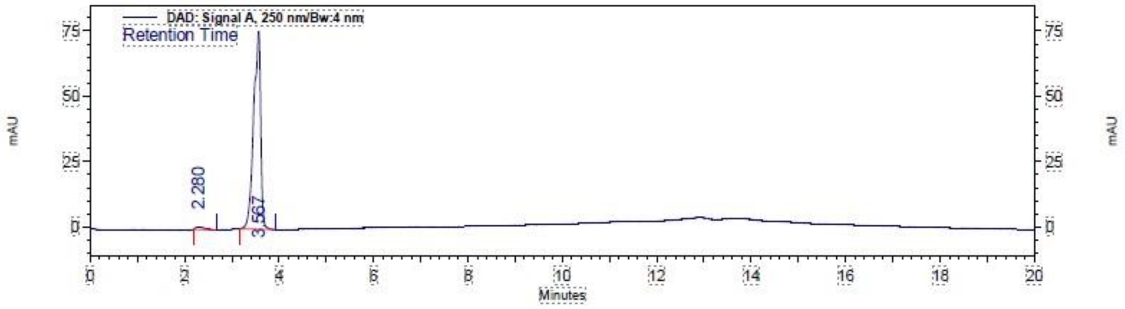

Figure 2: HPLC **Chromatogram of Thymol Aldehyde 2**

2.2 Computational analysis

In this study, to evaluate the ADME parameters of thymol and bioactive derivative (**3i)**, we have predicted the physicochemical parameters using a free web tool to SwissADME software. Molecular docking analysis was carried out for thymol (parent compound), and the most active compound **3i** was performed using AutoDock Tool 1.5.7 software using protein receptor (PDB id: 4HEF) of bacterial β-lactamase specimen of *P. aeruginosa* as previously reported in this paper (Swain et al.,2019).The interaction of the protein-ligand complex was visualized by Discovery Studio Visualizer software.

The 3D geometries of all the chiral molecules were optimized using Gaussian 09W programme and analyzed via GaussView 6 (Frisch et al.,2009). The DFT calculations were performed at hybrid exchange correlation functionals B3LYP (Sirianni et al., 2018) and basis set 6–311G (d,p) in the gas phase.

3. Characterization Data of Compounds

Thymol (1)

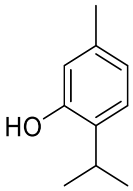

^{1}H NMR (400 MHz, $CDCl_3$) δ 7.11 (d, J = 7.8 Hz, 1H), 6.76 (d, J = 7.8 Hz, 1H), 6.60 (d, J = 0.9 Hz, 1H), 3.19 (dt, J = 13.8, 6.9 Hz, 1H), 2.30 (s, H), 1.27 (d, J = 6.9 Hz, 6H). ^{13}C NMR (101 MHz, $CDCl_3$) δ 152.55, 136.61, 131.32, 126.23, 121.63, 115.99, 26.71, 22.69, 20.88.

4-hydroxy-5-isopropyl-2-methylbenzaldehyde (Thymol aldehyde) (2):

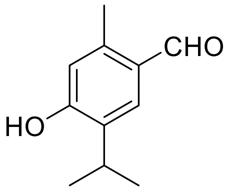

White amorphous solid (850.0 mg, 71% yield); ^{1}H NMR (400 MHz, $CDCl_3$) δ 10.11 (s, 1H), 7.68 (s, 1H), 6.64 (s, 1H), 3.21 (dt, J = 13.8, 6.9 Hz, 1H), 2.59 (s, 3H), 1.27 (d, J = 6.9 Hz, 6H). ^{13}C NMR (101 MHz, $CDCl_3$) δ 191.7, 158.1, 141.0, 132.8, 131.3, 127.7, 118.2, 26.7, 22.4, 19.01; HR-MS *m/z* 179.1073 $[M+H]^+$ (calcd for $C_{11}H_{14}O_2$, 178.0994)

Diethyl 4-(4-hydroxy-5-isopropyl-2-methylphenyl)-2,6-dimethyl-1,4 dihydropyridine-3,5-dicarboxylate (3a):

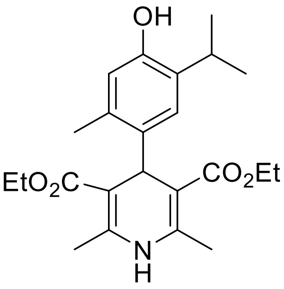

^{1}H NMR (400 MHz, $CDCl_3$) δ 7.04 – 6.96 (m, 1H), 6.27 (s, 1H), 5.02 – 4.93 (m, 1H), 4.02 (dddd, *J* = 9.2, 10.5, 5.4, 2.7 Hz, 4H), 3.06 – 2.93 (m, 1H), 2.40 – 2.13 (m, 9H), 1.18 (d, *J* = 2.0 Hz, 6H), 1.07 (d, *J* = 6.9 Hz, 6H); ^{13}C NMR (101 MHz, $CDCl_3$) δ 168.24, 151.09, 143.31, 139.28, 133.76, 131.58, 127.58, 116.35, 105.11, 59.81, 35.60, 29.71, 26.78, 22.63, 19.60, 18.86, 14.38; MS $[M+H]^-$ *m/z* 400.0995 (calcd for $C_{23}H_{31}NO_5$, 401.2202), Chemical Formula: $C_{23}H_{31}NO_5$

Dimethyl 4-(4-hydroxy-5-isopropyl-2-methylphenyl)-2,6-dimethoxy-1,4-dihydropyridine-3,5-dicarboxylate (3b):

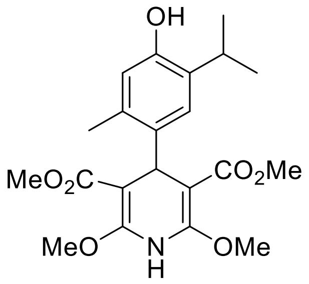

White amorphous solid; ^{1}H NMR (400 MHz, $CDCl_3$) (71mg, 62% yield) *δ* ^{1}H NMR (400 MHz, $CDCl_3$) δ 7.89 (s, 1H), 7.13 (s, 1H), 6.47 (s, 1H), 4.06 (q, *J* = 7.1 Hz, 1H), 3.76 (d, *J* = 5.2 Hz, 6H), 3.09 (dt, *J* = 13.7, 6.9 Hz, 1H), 2.21 (s, 3H), 1.99 (s, 1H), 1.11 (d, *J* = 6.9 Hz, 6H). ^{13}C NMR (101 MHz, $CDCl_3$) *δ* 168.2, 165.1, 155.5, 142.3, 137.8, 132.7, 126.4, 117.3, 60.6, 52.7, 52.6, 26.4, 22.6, 19.4, 14.2; MS *m/z* 404.1993 $[M+H]^-$ (calcd for $C_{21}H_{27}NO_7$, 405.1788); Chemical Formula: $C_{21}H_{27}NO_7$

Dibenzyl 4-(4-hydroxy-5-isopropyl-2-methylphenyl)-2,6-dimethyl-1,4-dihydropyridine-3,5-dicarboxylate (3c):

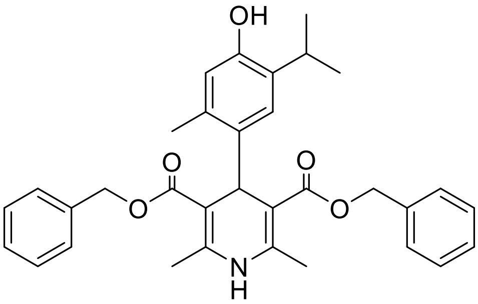

White amorphous solid (62mg, 42% yield) ^{1}H NMR (400 MHz, $CDCl_3$) δ7.23 (dd, *J* = 5.0, 2.0 Hz, 3H), 7.13 (dd, *J* = 6.9, 2.7 Hz, 5H), 7.03 (d, *J* = 9.0 Hz, 3H), 6.60 – 6.49 (m, 2H), 5.27 – 5.10 (m, 4H), 4.05 (q, *J* = 7.2 Hz, 2H), 2.30 (s, 5H), 2.25 (s, 2H), 2.17 (s, 1H), 1.98 (s, 1H), 1.11 (s, 6H).^{13}C NMR (101 MHz, $CDCl_3$) δ 168.5, 155.6, 143.6, 137.8, 132.8, 132.8, 131.8, 128.6, 128.5, 128.5, 128.4, 128.2, 128.0, 117.4, 105.1, 67.4, 31.3, 26.7, 22.5, 19.4, 19.4; HR-MS *m/z* 525.2471 $[M+H]^+$ (calcd. for $C_{33}H_{35}NO_5$, 525.2515); Chemical Formula: $C_{33}H_{35}NO_5$

Ethyl 4-(4-hydroxy-5-isopropyl-2-methylphenyl)-6-methyl-2-oxo-1,2,3,4-tetrahydropyrimidine-5-carboxylate (3d):

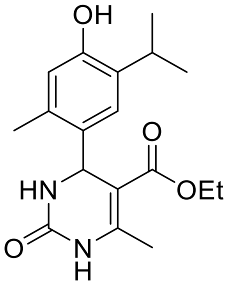

White amorphous solid (75.0 mg, 80 % yield); ^{1}H NMR (400 MHz, CD_3OD) *δ* 6.92 (s, 1H), 6.43 (s, 1H), 5.40 (d, *J* = 0.7 Hz, 1H), 3.89 (q, *J* = 7.1 Hz, 2H), 3.05 (dt, *J* = 13.8, 6.9 Hz, 1H), 2.30 – 2.17 (m, 6H), 1.08 – 0.92 (m, 9H). ^{13}C NMR (101 MHz, CD_3OD) δ 166.4, 153.4, 153.3, 146.6, 133.2, 133.1, 132.6, 124.8, 116.3, 101.1, 59.6, 51.4, 26.4, 21.7, 21.6, 17.3, 16.5, 13.1; HR-MS *m/z* 333.1813 $[M+H]^+$ (calcd. for $C_{18}H_{24}N_2O_4$, 332.1736)

Benzyl 4-(4-hydroxy-5-isopropyl-2-methylphenyl)-6-methyl-2-oxo-1,2,3,4-tetrahydropyrimidine-5-carboxylate (3e):

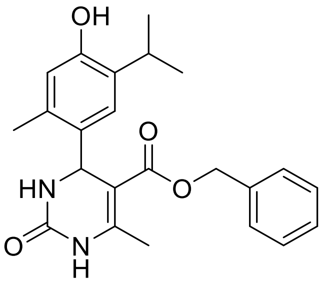

White amorphous solid (81 mg, 73% yield); ^{1}H NMR (400 MHz, CD_3OD) δ 7.14 – 7.06 (m, 3H), 6.92 (s, 1H), 6.83 – 6.78 (m, 2H), 6.39 (s, 1H), 5.41 (d, J = 0.7 Hz, 1H), 4.98 (d, J = 12.6 Hz, 1H), 3.05 (dt, J = 13.8, 6.9 Hz, 1H), 2.27 (d, J = 0.6 Hz, 3H), 2.05 (s, 3H), 1.94 (s, 3H), 1.00 (dd, J = 6.9, 2.5 Hz, 6H); ^{13}C NMR (101 MHz, CD_3OD) δ 167.8, 155.4, 155.1, 149.5, 138.2, 135.2, 135.2, 134.6, 129.9, 129.3, 129.1, 126.5, 118.4, 102.6, 67.1, 53.1, 28.4, 23.6, 23.5, 19.1, 18.5; HR-MS *m/z* 394.1842 $[M+H]^+$ (calcd. for $C_{23}H_{26}N_2O_4$, 394.1893)

4-(4-hydroxy-5-isopropyl-2-methylphenyl)-6-isopropoxy-5-(2,2,2- trifluoroacetyl)-3,4-dihydropyrimidin-2(1H)-one (3f):

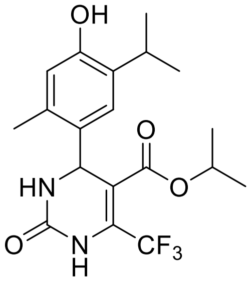

White amorphous solid (42.0 mg, 37% yield**);** ^{1}H NMR (400 MHz, CD_3OD) δ 7.71 – 7.46 (m, 1H), 6.47 (s, 1H), 3.65 – 3.59 (m, 2H), 3.25 (s, 1H), 2.18 (d, J = 10.6 Hz, 3H), 1.14 – 1.08 (m, 9H), 0.71 (t, J = 7.1 Hz, 3H). ^{13}C NMR (101 MHz, CD_3OD) δ 169.71, 168.07, 154.51, 153.94, 135.18, 132.13, 131.13, 128.51, 123.90, 104.78, 88.62, 67.80, 60.97, 38.75, 30.22, 28.74,

23.56, 22.65, 21.56.MS *m/z* 341.2481 $[M\text{-}CH_3COO]^-$ (calcd for $C_{19}H_{23}F_3N_2O_4$, 400.1610); Chemical Formula: $C_{19}H_{23}F_3N_2O_4$

5-(cyclopropanecarbonyl)-4-(4-hydroxy-5-isopropyl-2-methylphenyl)-6-methoxy-3,4-dihydropyrimidin-2(1H)-one (3g):

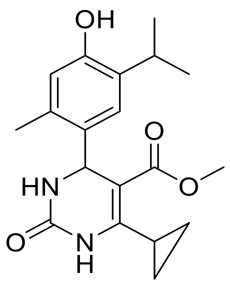

White amorphous solid (55mg, 56% yield); ^{1}H NMR (400 MHz, CD_3OD) δ 6.92 (s, 1H), 6.41 (s, 1H), 5.39 (s, 1H), 3.48 (s, 3H), 3.13 – 3.00 (m, 1H), 2.85 (s, 1H), 2.22 (s, 3H), 1.02 (dd, J = 6.9, 1.4 Hz, 6H), 0.92 – 0.76 (m, 4H). ^{13}C NMR (101 MHz, CD_3OD) δ 167.0, 153.4, 150.7, 133.1, 133.0, 132.6, 124.5, 116.4, 102.3, 51.2, 50.3, 26.4, 21.8, 21.6, 17.2, 11.2. MS *m/z* 343.2003, $[M\text{-}H]^-$ (calcd for $C_{19}H_{24}N_2O_4$, 344.1736); Chemical Formula: $C_{19}H_{24}N_2O_4$

Ethyl 4-(4-hydroxy-5-isopropyl-2-methylphenyl)-6-methyl-2-thioxo-1,2,3,4-tetrahydropyrimidine-5-carboxylate (3h):

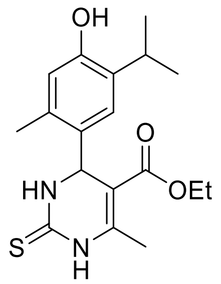

White amorphous solid (27mg, 62% yield); ^{1}H NMR (400 MHz, CD_3OD) δ 7.53 (s, 1H), 7.03 (s, 1H), 6.45 (s, 1H), 3.79 – 3.59 (m, 2H), 3.21 (d, J = 1.5 Hz, 1H), 3.17 – 3.08 (m, 1H), 2.19 (d, J = 2.7 Hz, 3H), 1.24 – 1.03 (m, 9H), 0.71 (t, J = 7.1 Hz, 3H). ^{13}C NMR (101 MHz, CD_3OD) δ 173.66, 168.13, 158.51, 155.01, 151.02, 139.07, 111.87, 108.78, 102.01, 100.20, 92.57,

64.84, 54.78, 33.29, 30.58, 25.67, 25.43, 16.33. MS *m/z* 365.0542 $[M+NH_3]^-$ (calcd for $C_{18}H_{24}N_2O_3S$, 348.1508); Chemical Formula: $C_{18}H_{24}N_2O_3S$

Ethyl 4-(4-hydroxy-5-isopropyl-2-methylphenyl)-2-imino-6-methyl-1,2,3,4-tetrahydropyrimidine-5-carboxylate (3i):

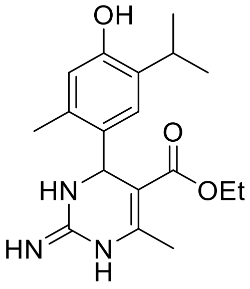

White amorphous solid (65.0 mg, 69% yield; ^{1}H NMR (400 MHz, CD_3OD) 7.52 (s, 1H), 6.54 (s, 1H), 4.56 (bs, 1H), 3.13 (q, *J* = 8.0 Hz, 2H), 3.11 (m, 1H), 2.42 (s, 6H), 1.13 (s, 3H), 1.11 (s, 6H).

^{13}C NMR (101 MHz, CD_3OD) 160.29, 154.06, 140.84, 133.06, 131.30, 131.00, 126.48, 117.35, 102.45, 61.26, 51.55, 26.38, 21.77, 21.37,21.37, 17.93, 17.16. MS *m/z* 330.9531 $[M\text{-}H^+]^-$ (calcd for $C_{18}H_{25}N_3O_3$, 331.1896); Chemical Formula: $C_{18}H_{25}N_3O_3$.

4. NMR spectra

^{1}H spectra of thymol (1):

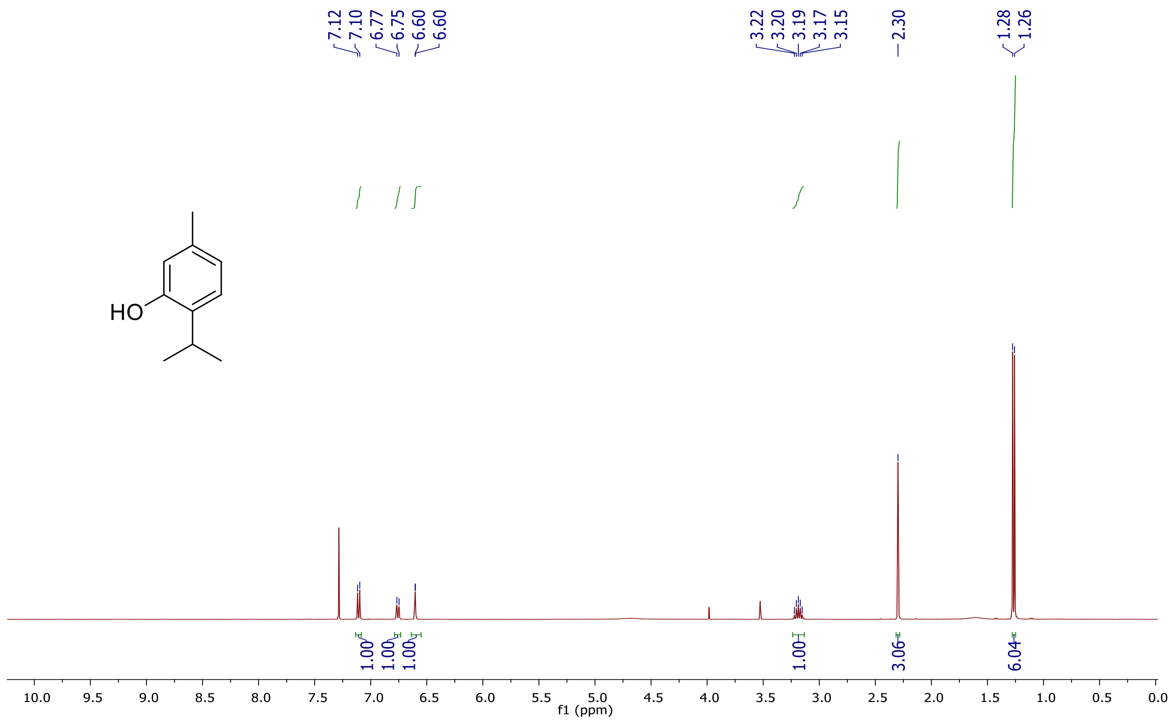

^{13}C spectra of thymol (1):

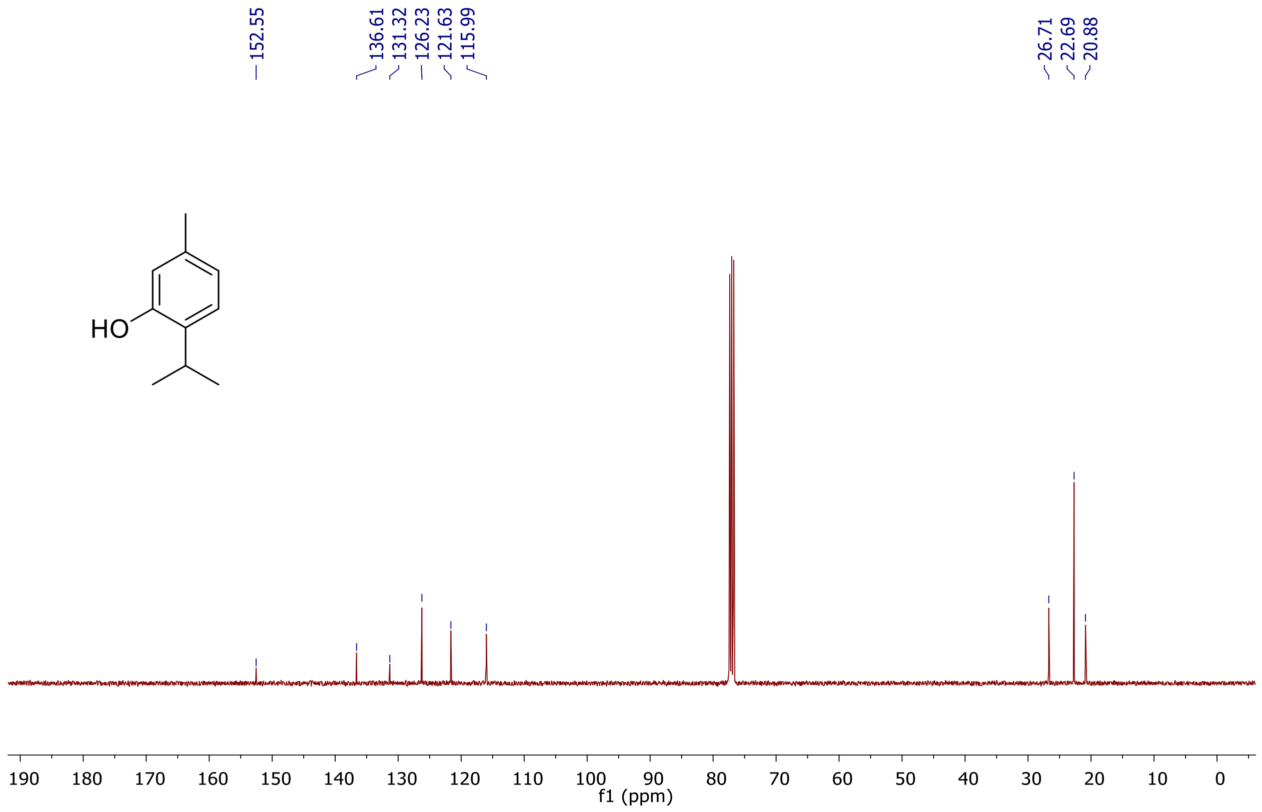

^{1}H spectra of thymol Aaldehyde (2):

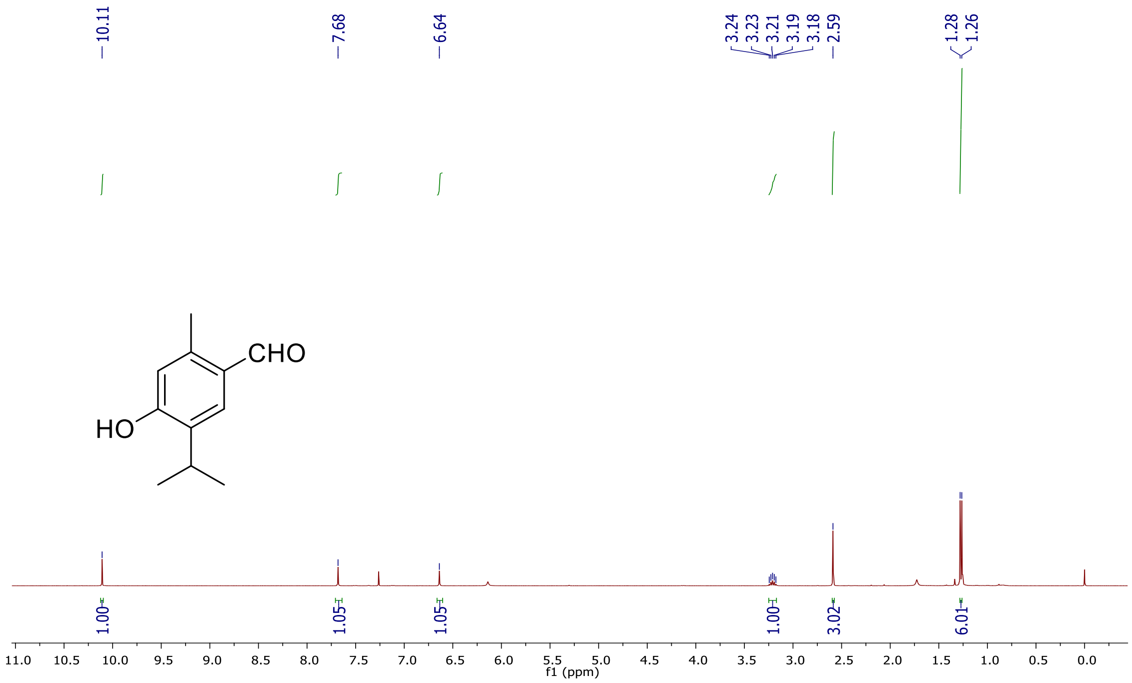

^{13}C spectra of thymol aldehyde (2):

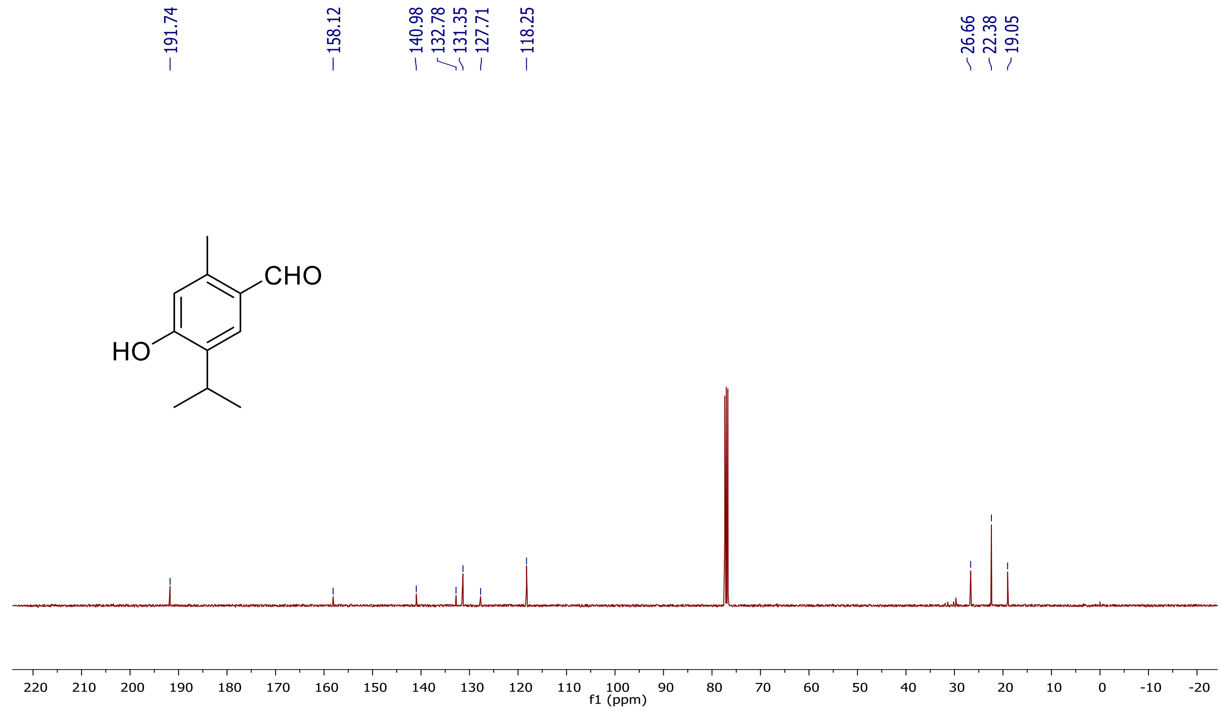

^{1}H spectra of compound 3a:

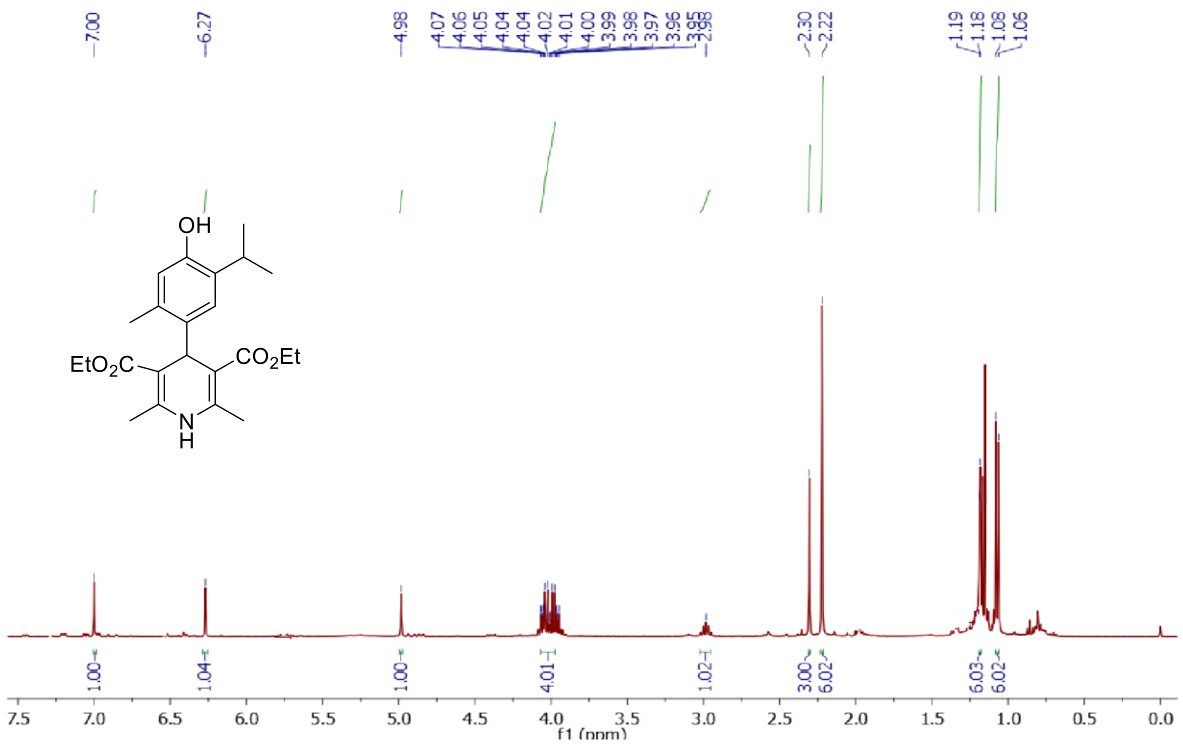

^{13}C spectra of compound 3a:

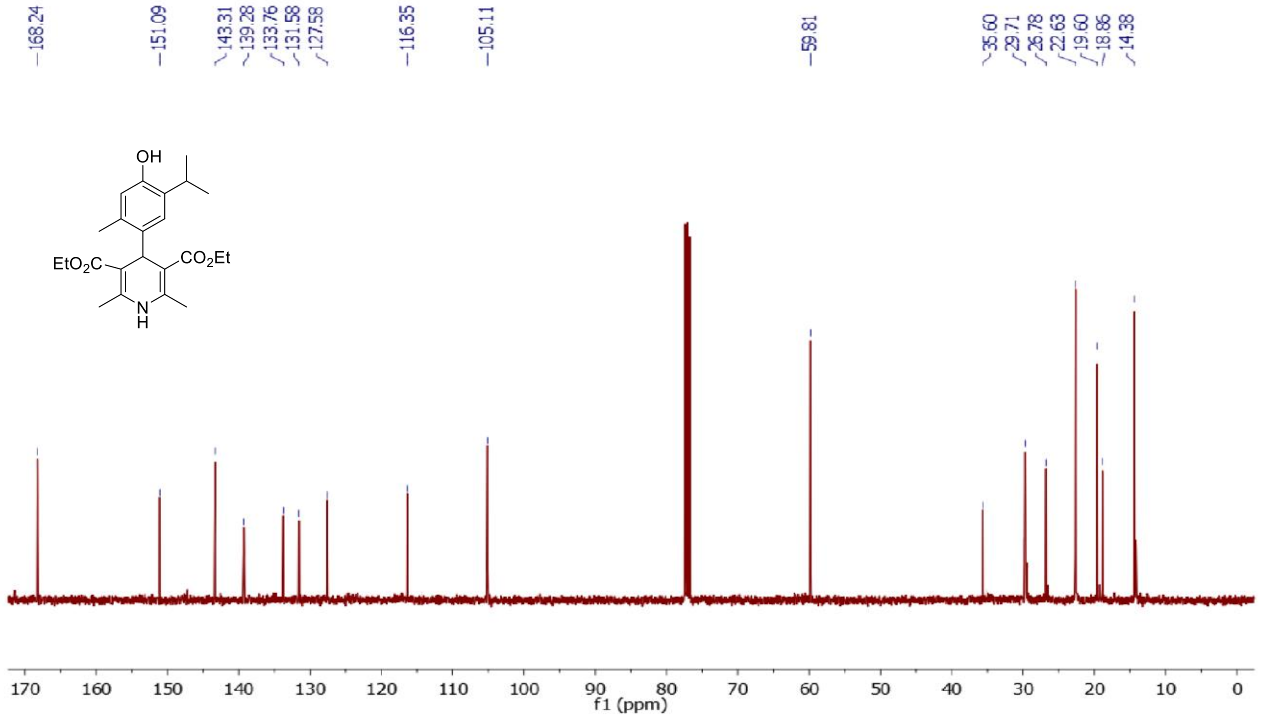

^{1}H spectra of compound 3b:

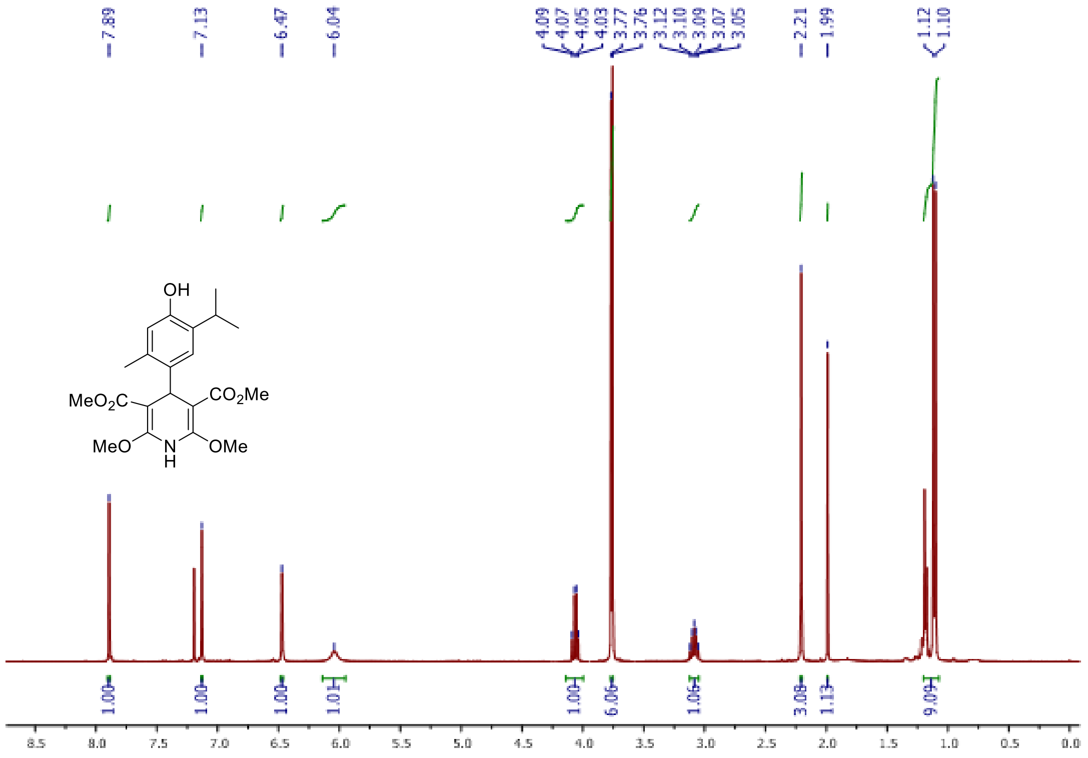

^{13}C spectra of compound 3b

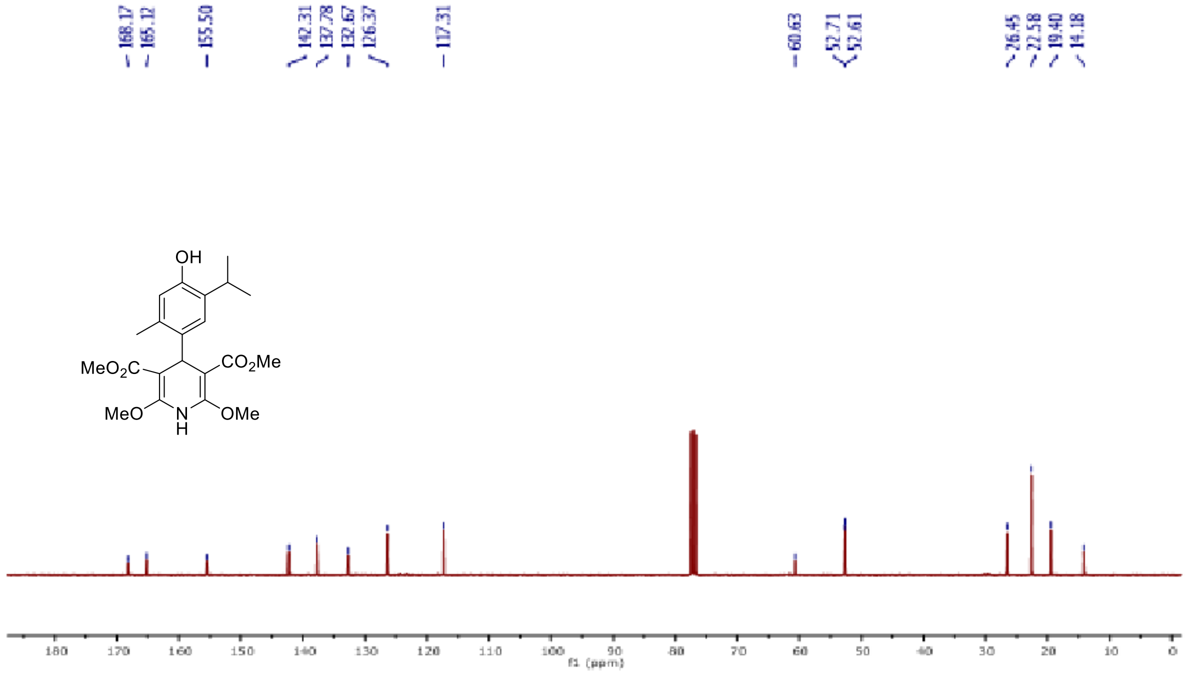

^{1}H spectra of compound 3c

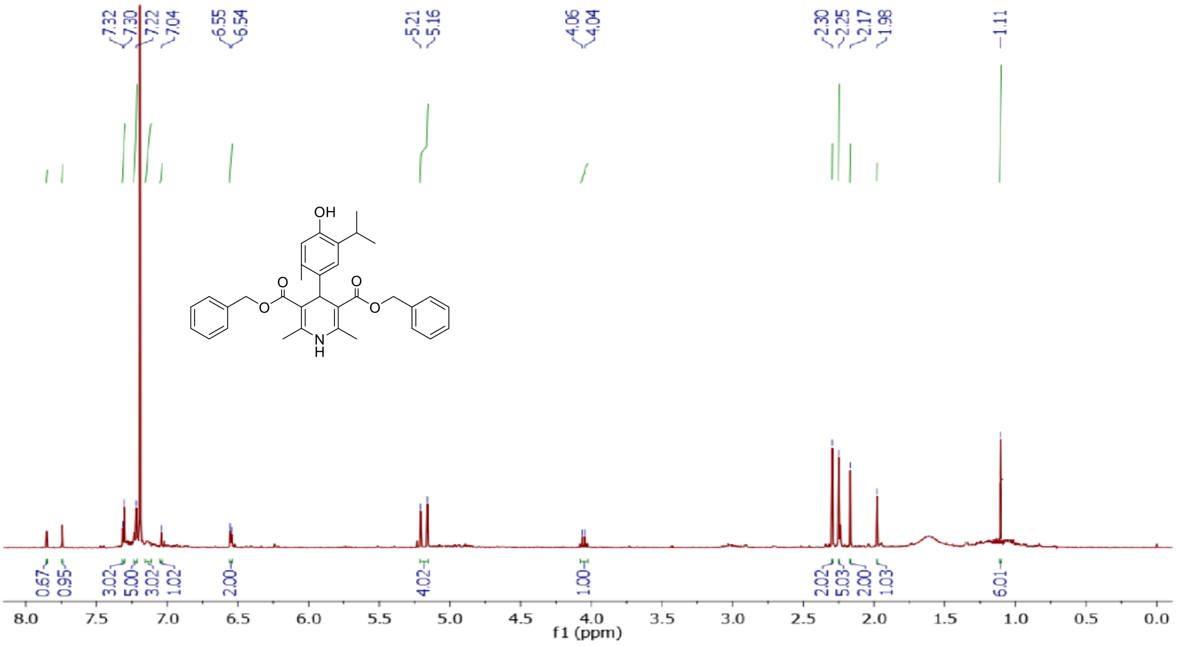

^{13}C spectra of compound 3c

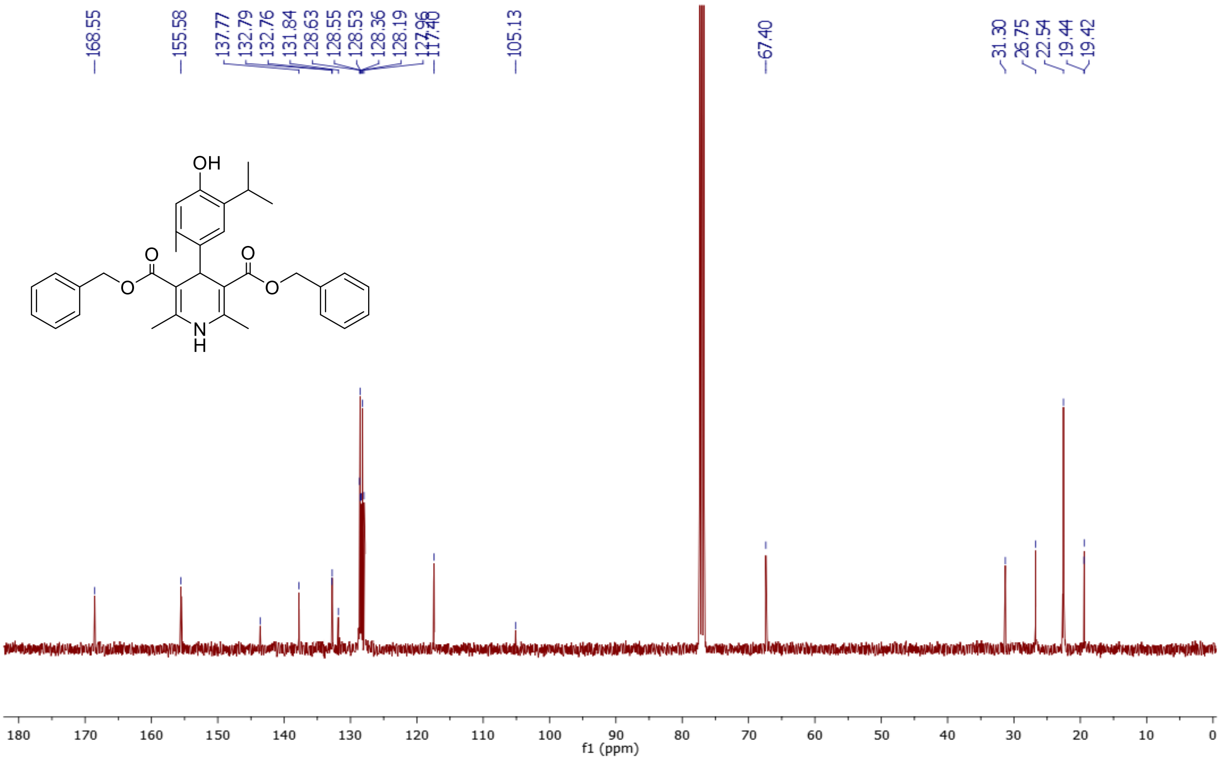

^{1}H spectra of compound 3d:

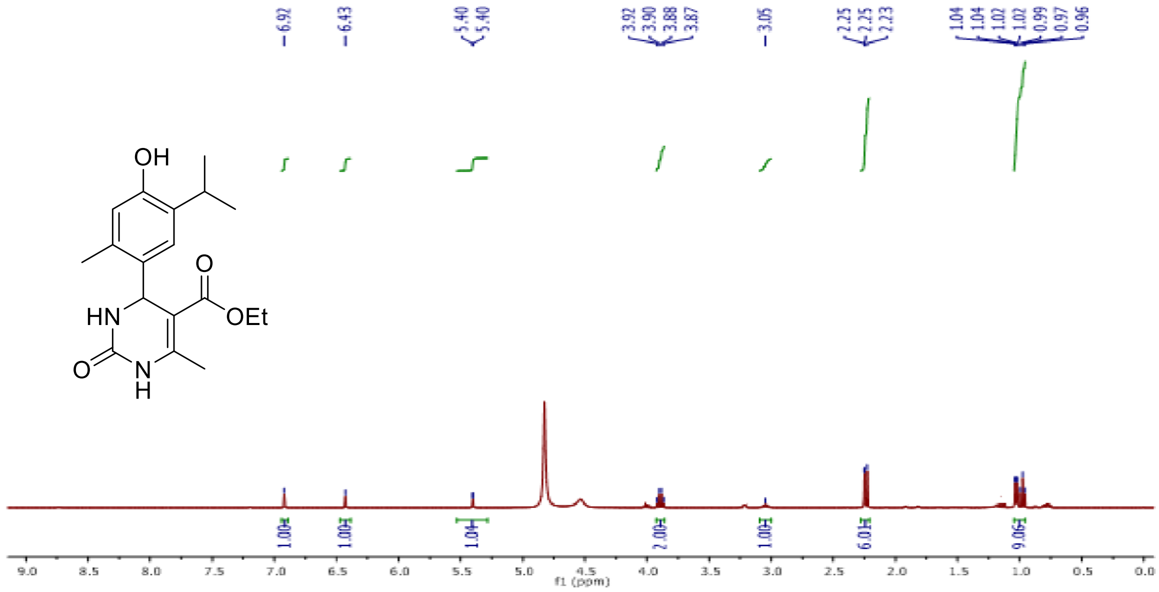

^{13}C spectra of compound 3d

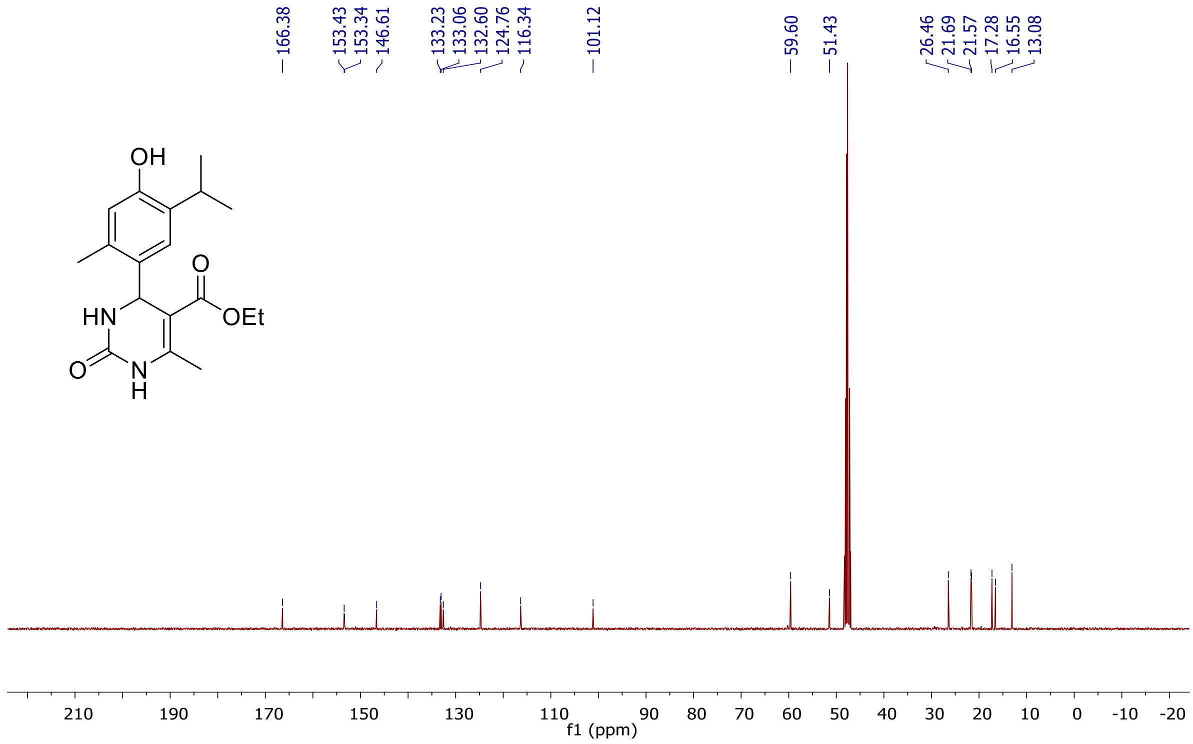

^{1}H spectra of compound 3e

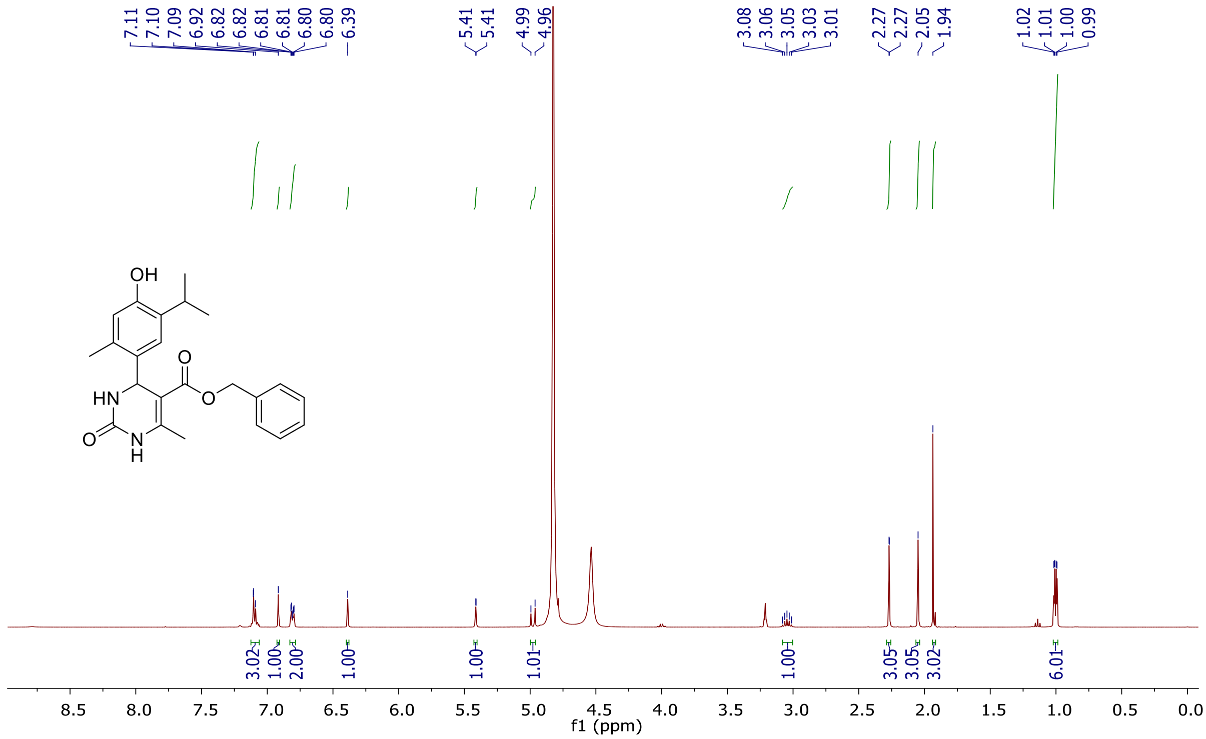

^{13}C spectra of compound 3e

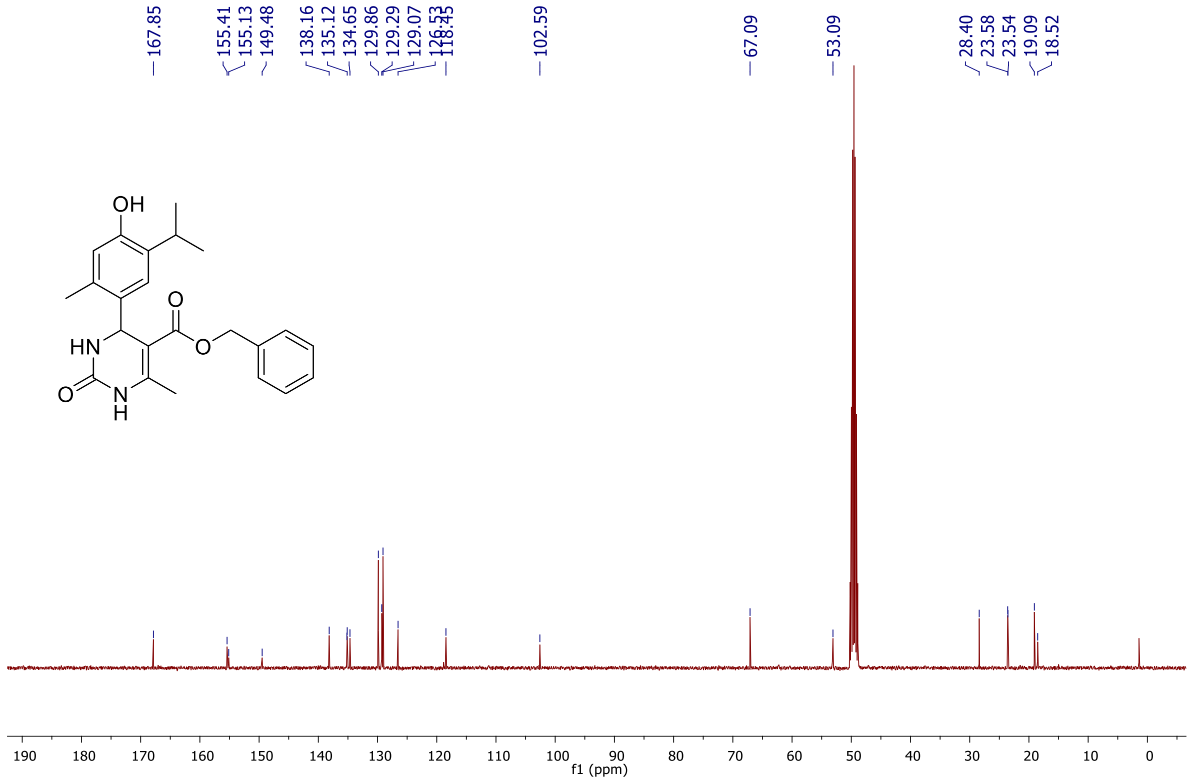

^{1}H spectra of compound 3f

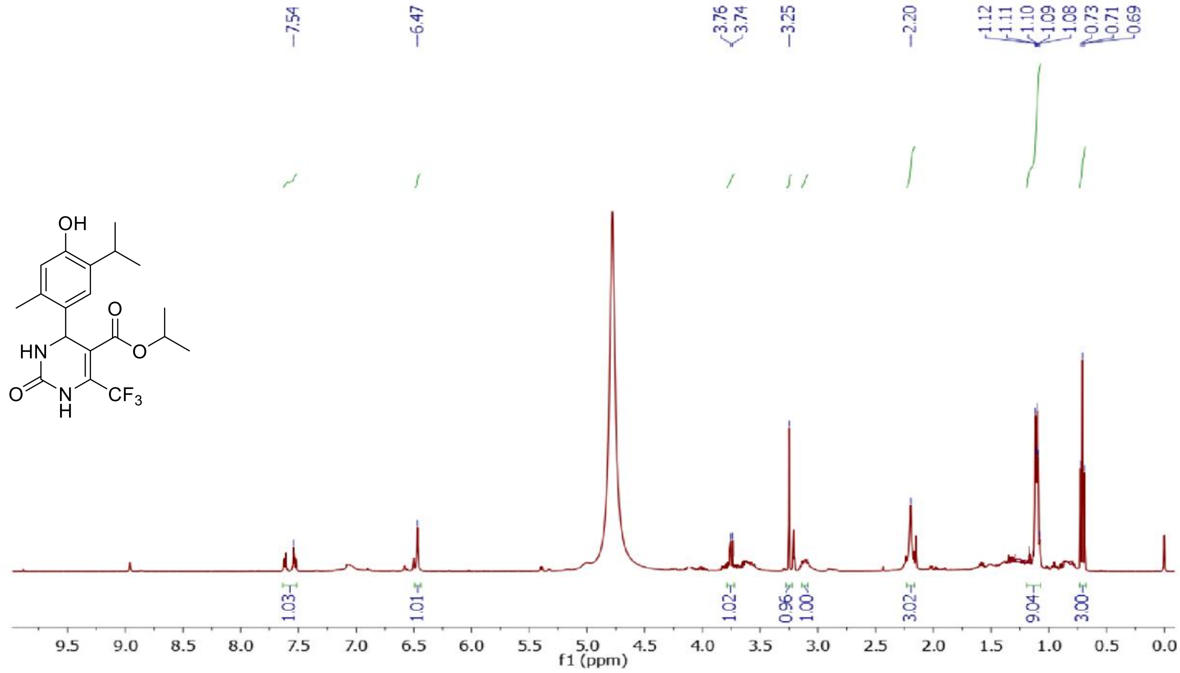

^{13}C spectra of compound 3f

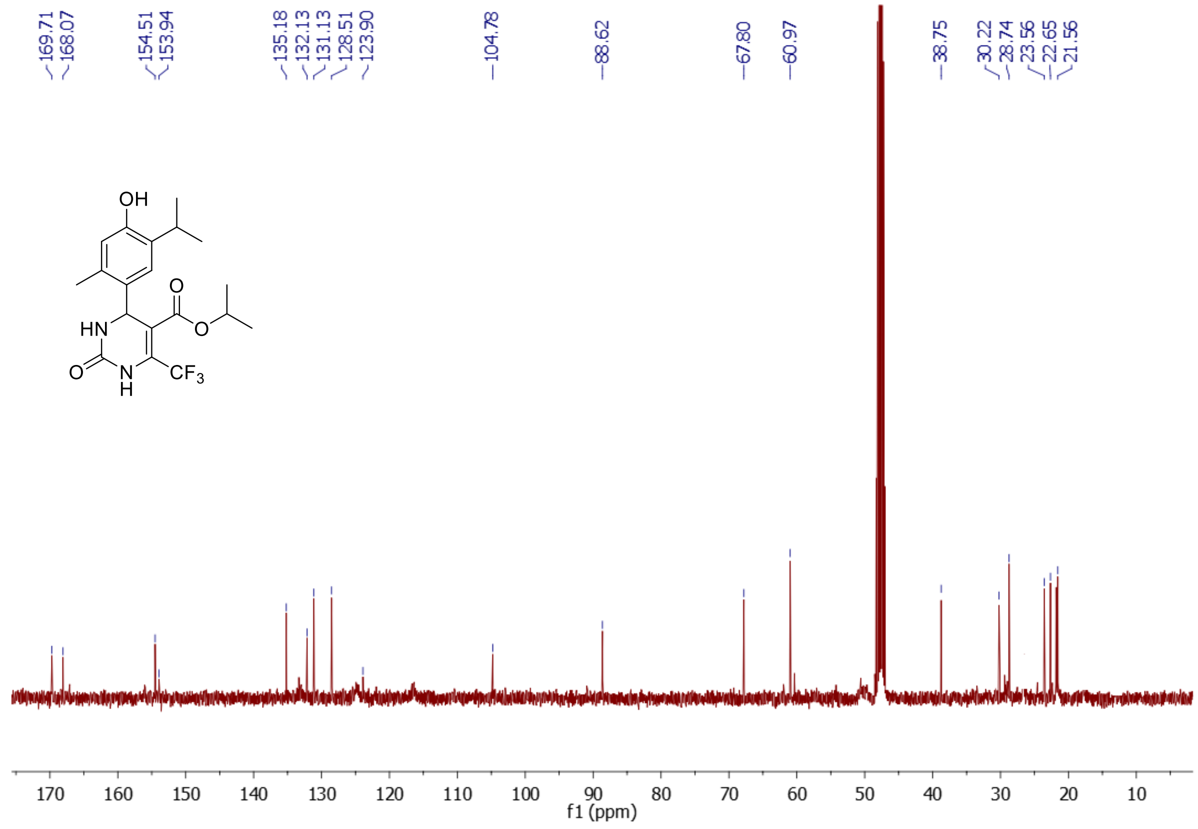

^{1}H spectra of compound 3g

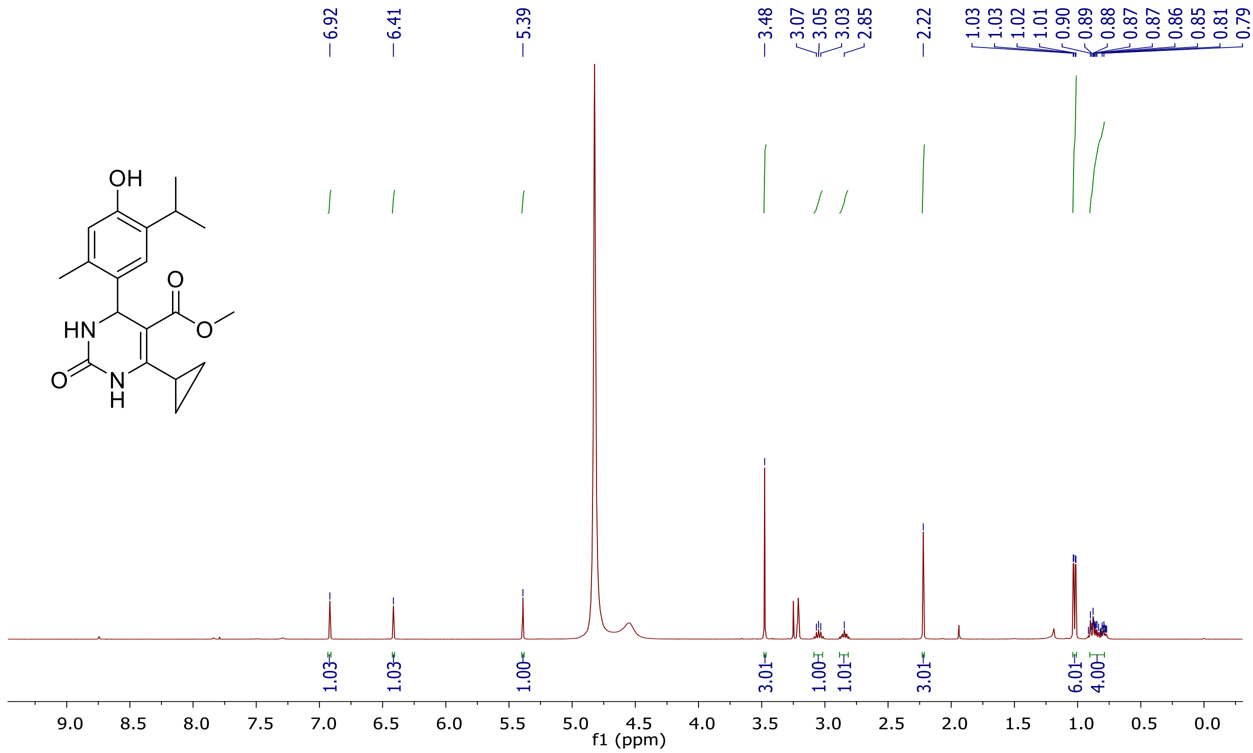

^{13}C spectra of compound 3g

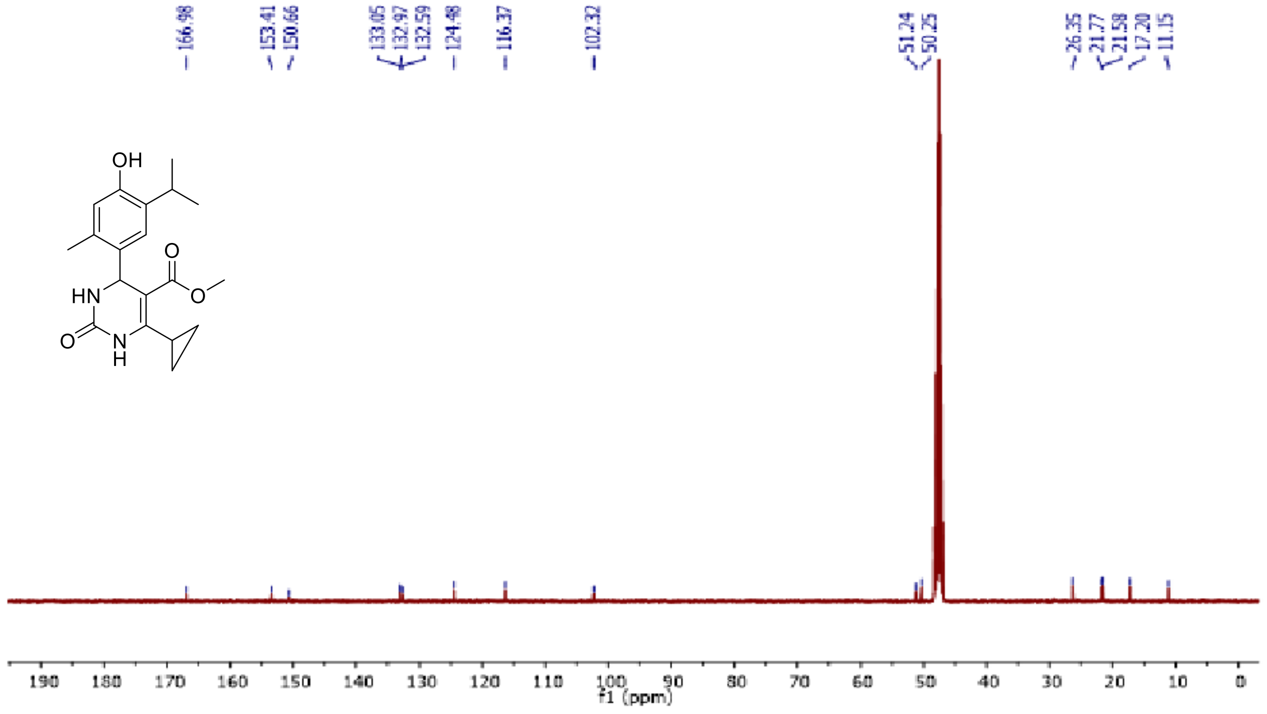

^{1}H spectra of compound 3h:

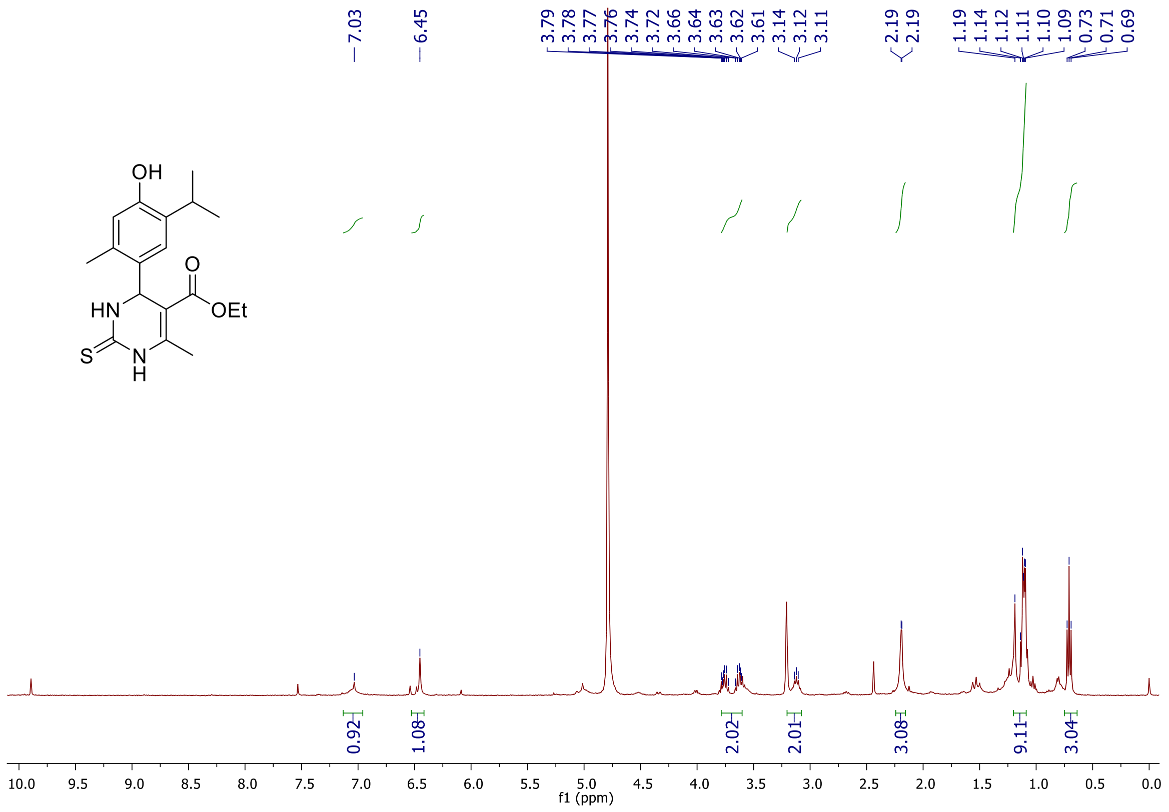

^{13}C spectra of compound 3h

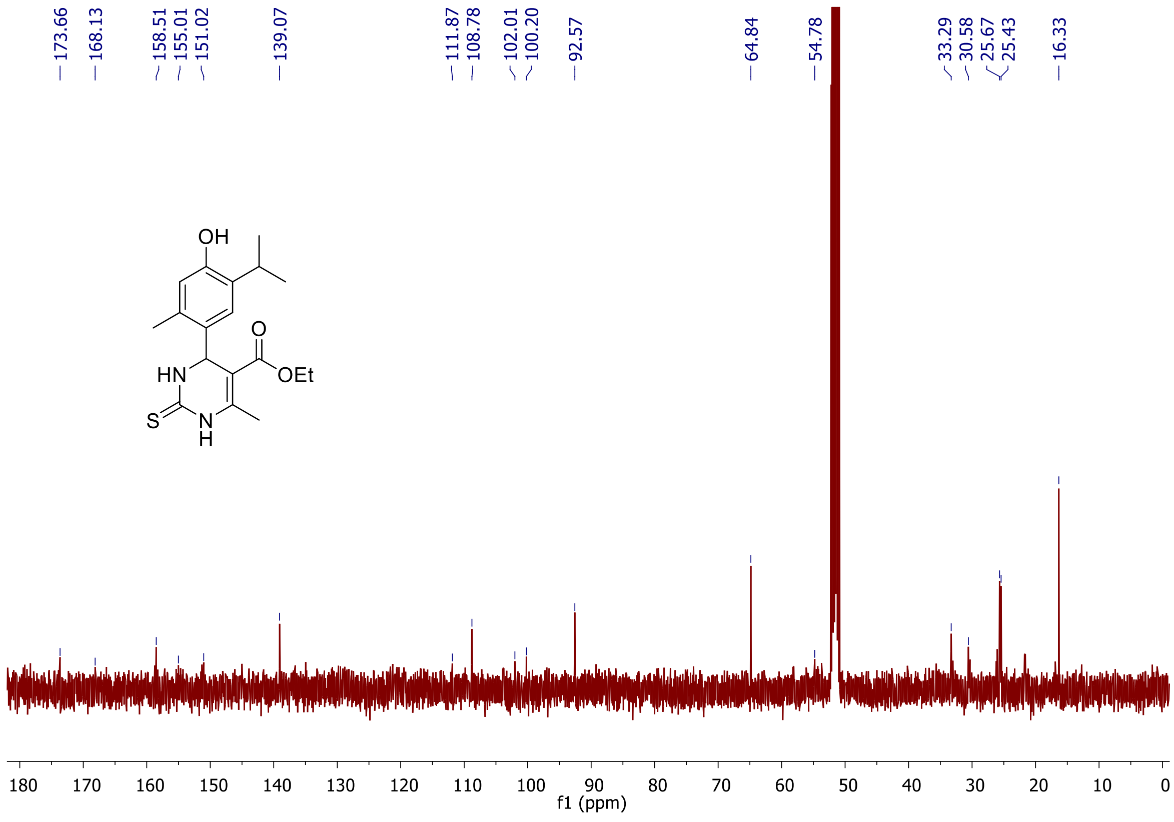

^{1}H spectra of compound 3i

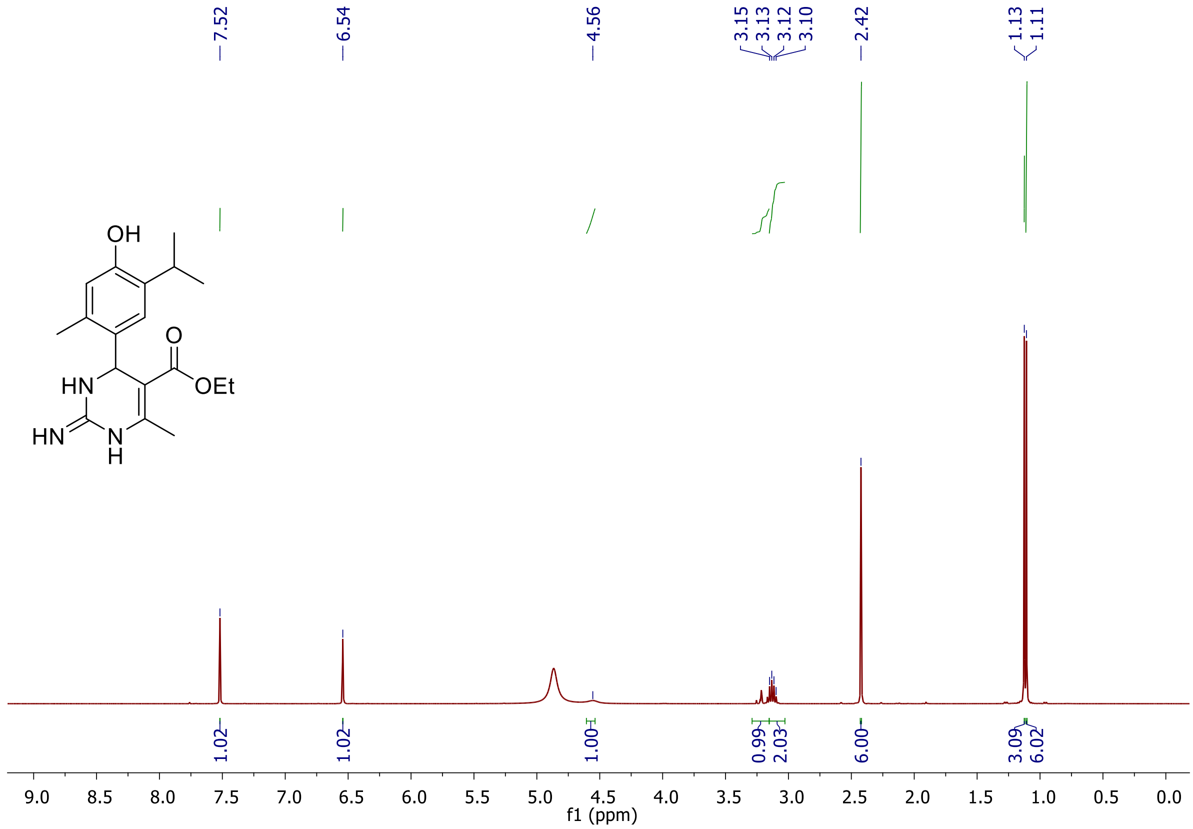

^{13}C spectra of compound 3i

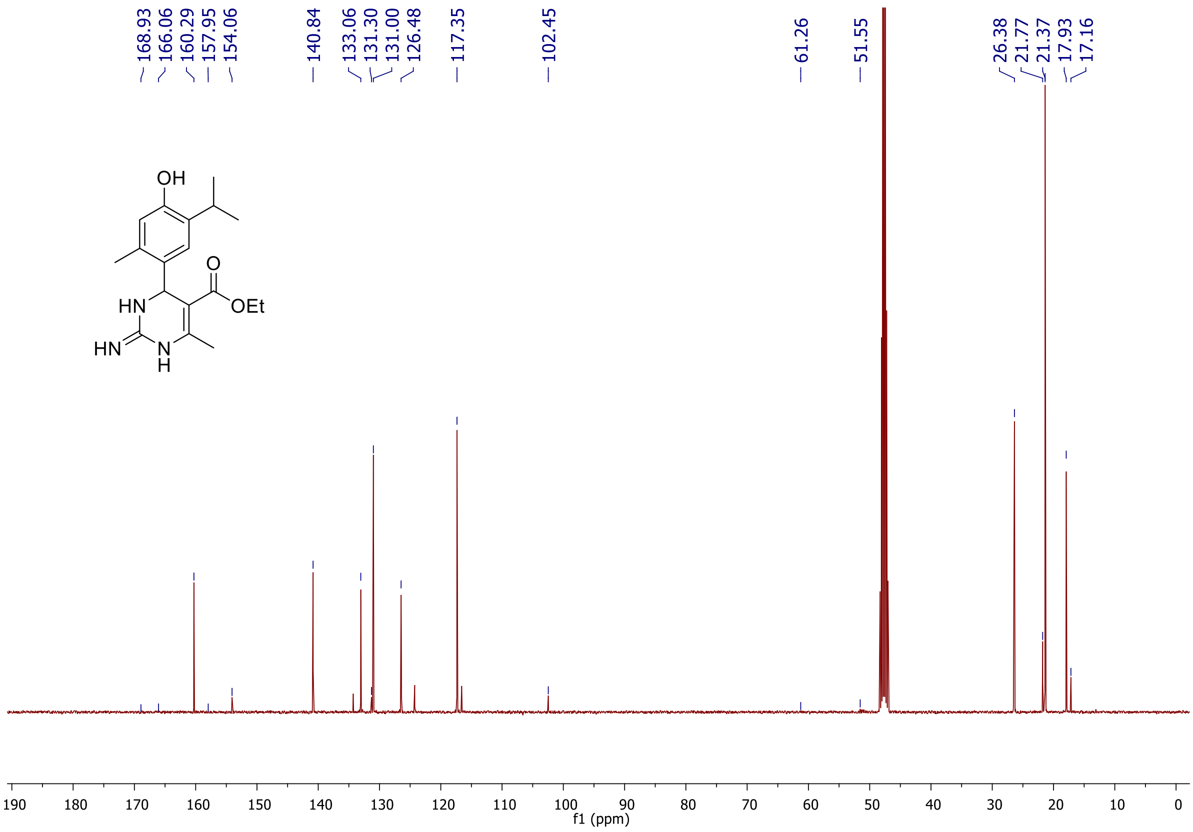

5. Crystal Experiment

Single crystal X-ray diffraction:

Single crystals, suitable for X-ray diffraction analysis, were grown by slow evaporation of a concentrated solution of the compounds. Data were collected on a Bruker Apex II CCD diffractometer with MoKα (λ = 0.71073) radiation. Preliminary lattice parameters and orientation matrices were obtained from three sets of frames. The full data were collected using the ω and ϕ scan methods with a frame width of 0.5°. Data were processed with the SAINT+ program for reduction and cell refinement. Multi-scan absorption corrections were applied by using the SADABS program for the area detector. The structures were solved by SHELXT[1] and refined with SHELXL(N.K. Sebbar. et al.,2015) using the Olex2 program(V.O.Dolomanov.et al.,2019). The CIFs are submitted into CCDC *2287405* and can be obtained through https://summary.ccdc.cam.ac.uk/structure-summary-form.

6. Supplementary Figure 3: Theoretically optimized structures of the molecules

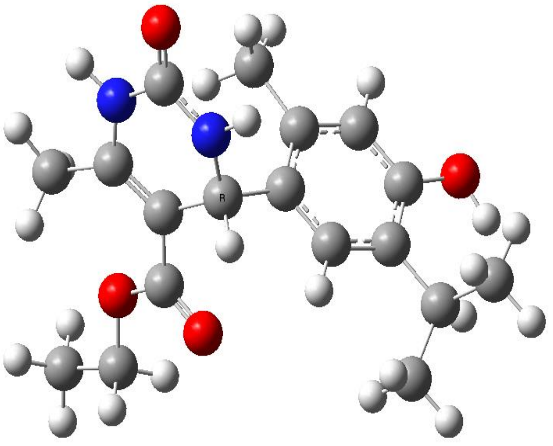

3d (*R-isomer*)

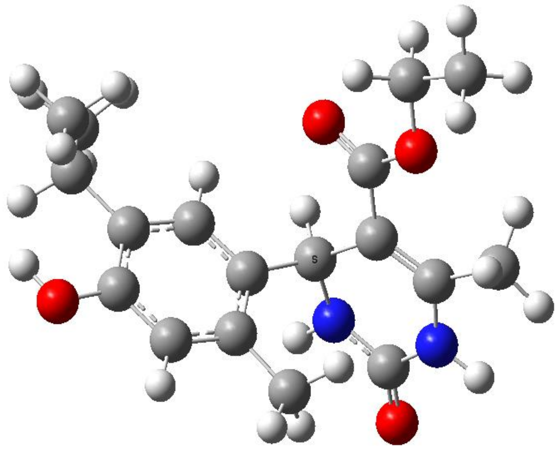

3d (*S-isomer*)

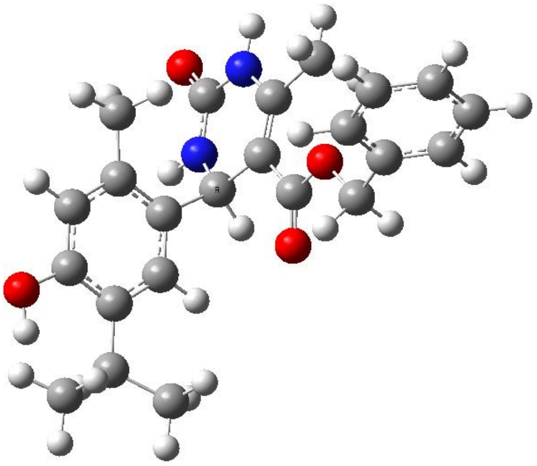

3e (*R-isomer*)

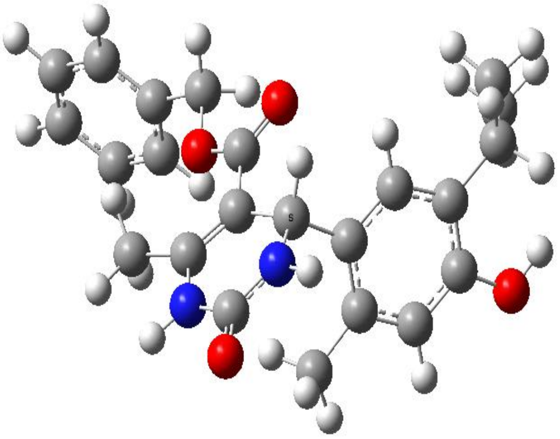

3e (*S-isomer*)

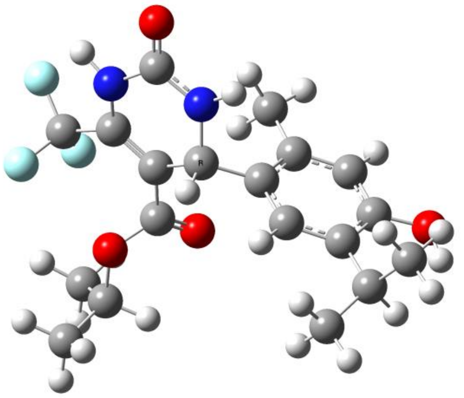

3f (*R-isomer*)

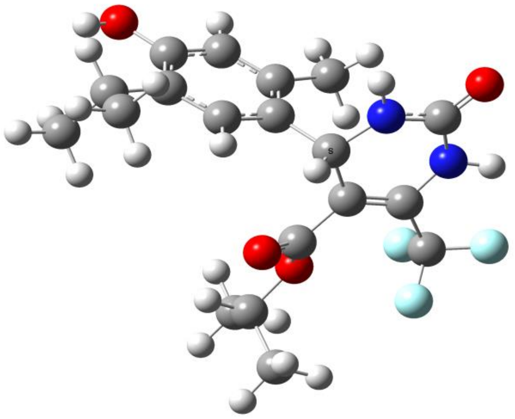

3f (*S-isomer*)

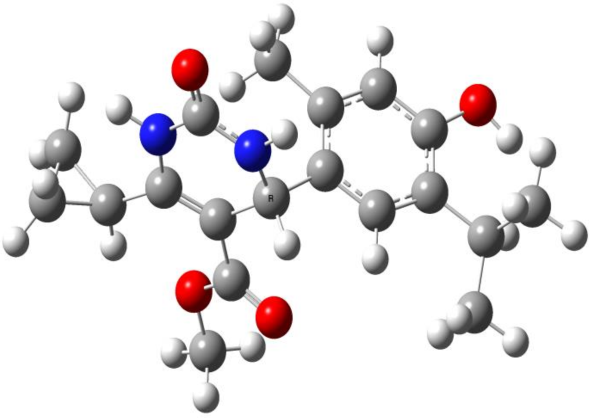

3g (*R-isomer*)

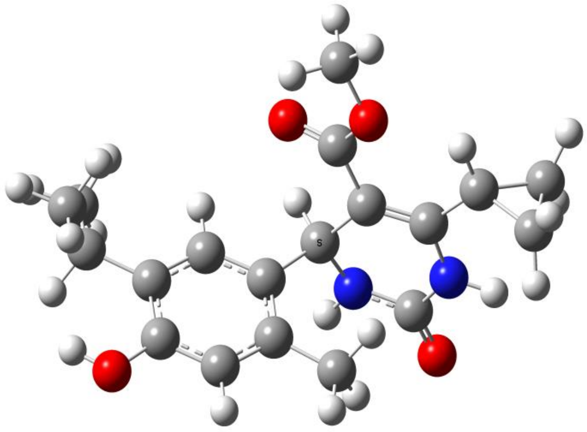

3g (*S-isomer*)

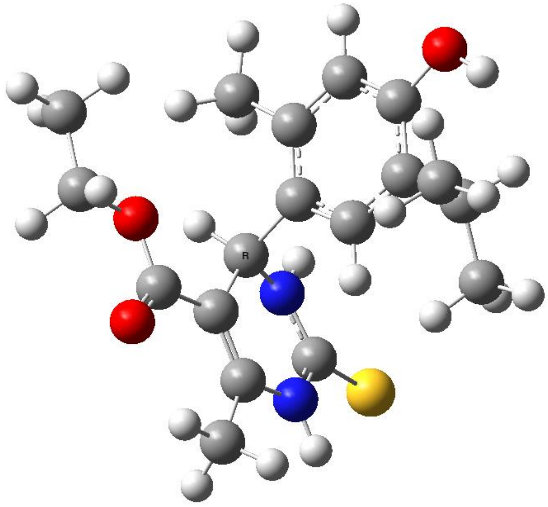

3h (*R-isomer*)

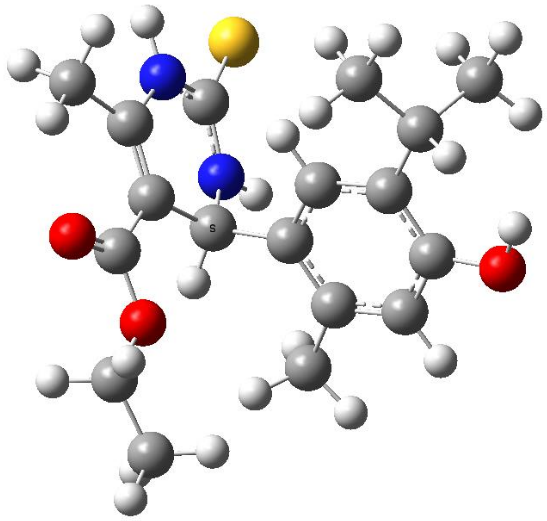

3h (*S-isomer*)

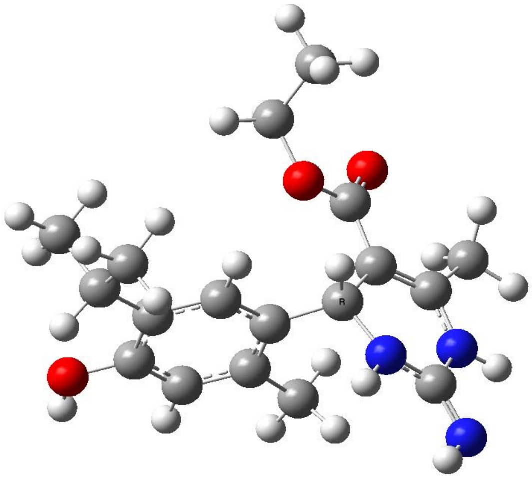

3i (*R-isomer*)

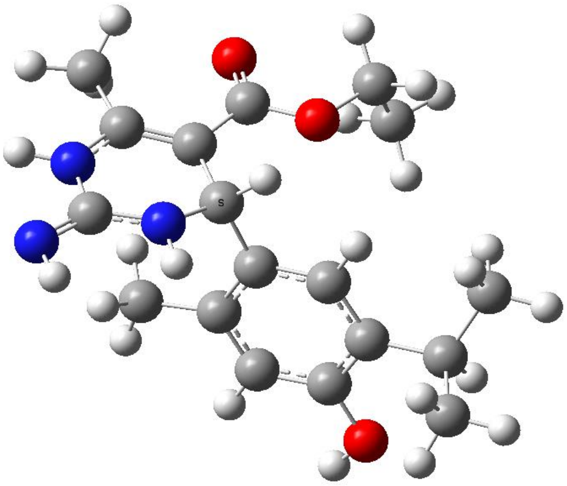

3i (*S-isomer*)

7. Biology Experimental Section:

Antibacterial properties of thymol derivatives:- we have added the MBC value of compound 3i which is represented in Figure 4.

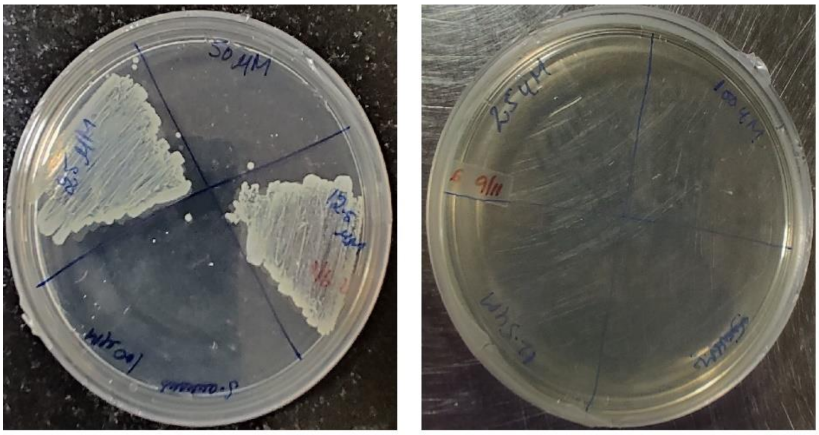

MRSA MBC [50μM] P. aeruginosa MBC [12.5μM]

Figure. 4 MBC value of compound 3i

TABLE 1: Synergy FIC index between standard antibiotic (Vancomycin) and compound (3j) against *P. aeruginosa*

S. No.	Compound	MIC value (μM) In combination	MIC value (μM) single	FICI	RESULT
1	3i	6.25	12.5	0.50	Partial synergy
	Vancomycin	0.07	12.5		
2	3i	6.25	12.5	0.50	Synergy
	Vancomycin	0.03	12.5		

TABLE 2: Indifference/Additive FIC index between standard antibiotic (Vancomycin) and compound (3j) against *P. aeruginosa*

S. No.	Compound	MIC value (µM) In combination	MIC value (µM) single	FICI	RESULT
1	3i	25	12.5	4	Indifference/ Additive
	Vancomycin	25	12.5		
2	3i	25	12.5	3	Indifference/ Additive
	Vancomycin	12.5	12.5		
3	3i	25	12.5	2.5	Indifference/ Additive
	Vancomycin	6.25	12.5		
4	3i	25	12.5	2.2	Indifference/ Additive
	Vancomycin	3.1	12.5		
5	3i	25	12.5	2.1	Indifference/ Additive
	Vancomycin	1.5	12.5		
6	3i	25	12.5	2.0	Indifference/ Additive
	Vancomycin	0.7	12.5		
7	3i	25	12.5	2.0	Indifference/ Additive
	Vancomycin	0.3	12.5		
8	3i	25	12.5	2.0	Indifference/ Additive
	Vancomycin	0.1	12.5		
9	3i	25	12.5	2.0	Indifference/ Additive
	Vancomycin	0.07	12.5		
10	3i	25	12.5	2.0	Indifference/ Additive
	Vancomycin	0.03	12.5		
11	3i	12.5	12.5	5	Indifference/ Additive
	Vancomycin	50	12.5		
12	3i	12.5	12.5	3	Indifference/ Additive
	Vancomycin	25	12.5		
13	3i	12.5	12.5	2	Indifference/ Additive
	Vancomycin	12.5	12.5		
14	3i	12.5	12.5	1.5	Indifference/ Additive
	Vancomycin	6.25	12.5		
15	3i	12.5	12.5	1.2	Indifference/ Additive
	Vancomycin	3.1	12.5		
16	3i	12.5	12.5	1.1	Indifference/ Additive
	Vancomycin	1.5	12.5		
17	3i	12.5	12.5	1.0	Indifference/ Additive
	Vancomycin	0.7	12.5		
18	3i	12.5	12.5	1.0	Indifference/ Additive
	Vancomycin	0.3	12.5		
19	3i	12.5	12.5	1.0	Indifference/ Additive
	Vancomycin	0.1	12.5		
20	3i	12.5	12.5	1.0	Indifference/ Additive
	Vancomycin	0.07	12.5		
21	3i	12.5	12.5	1.0	Indifference/ Additive
	Vancomycin	0.03	12.5		
22	3i	6.25	12.5	4.5	Indifference/ Additive
	Vancomycin	50	12.5		

23	3i	6.25	12.5	2.5	Indifference/ Additive
	Vancomycin	25	12.5		
24	3i	6.25	12.5	1.5	Indifference/ Additive
	Vancomycin	12.5	12.5		
25	3i	6.25	12.5	1	Indifference/ Additive
	Vancomycin	6.25	12.5		
26	3i	6.25	12.5	0.7	Indifference/ Additive
	Vancomycin	3.1	12.5		
27	3i	6.25	12.5	0.6	Indifference/ Additive
	Vancomycin	1.5	12.5		
28	3i	6.25	12.5	0.55	Indifference/ Additive
	Vancomycin	0.7	12.5		
29	3i	6.25	12.5	0.52	Indifference/ Additive
	Vancomycin	0.3	12.5		
30	3i	6.25	12.5	0.51	Indifference/ Additive
	Vancomycin	0.1	12.5		
31	3i	3.12	12.5	4.25	Indifference/ Additive
	Vancomycin	50	12.5		
32	3i	3.12	12.5	2.25	Indifference/ Additive
	Vancomycin	25	12.5		
33	3i	3.12	12.5	1.25	Indifference/ Additive
	Vancomycin	12.5	12.5		
34	3i	3.12	12.5	0.75	Indifference/ Additive
	Vancomycin	6.25	12.5		
35	3i	1.5	12.5	4.1	Indifference/ Additive
	Vancomycin	50	12.5		
36	3i	1.5	12.5	2.1	Indifference/ Additive
	Vancomycin	25	12.5		
37	3i	1.5	12.5	1.1	Indifference/ Additive
	Vancomycin	12.5	12.5		
38	3i	1.5	12.5	0.6	Indifference/ Additive
	Vancomycin	6.25	12.5		
39	3i	0.7	12.5	4.0	Indifference/ Additive
	Vancomycin	50	12.5		
40	3i	0.7	12.5	2.0	Indifference/ Additive
	Vancomycin	25	12.5		
41	3i	0.7	12.5	1.0	Indifference/ Additive
	Vancomycin	12.5	12.5		
42	3i	0.7	12.5	0.55	Indifference/ Additive
	Vancomycin	6.25	12.5		
43	3i	0.3	12.5	4.0	Indifference/ Additive
	Vancomycin	50	12.5		
44	3i	0.3	12.5	2.0	Indifference/ Additive
	Vancomycin	25	12.5		
45	3i	0.3	12.5	1.0	Indifference/ Additive
	Vancomycin	12.5	12.5		
46	3i	0.3	12.5	0.52	Indifference/ Additive
	Vancomycin	6.25	12.5		

TABLE 3: Synergy FIC index between standard antibiotic (Vancomycin) and compound (3i) against MRSA (Methicillin-Resistant *Staphylococcus aureus*)

S. No.	Compound	MIC value (µM) in combination	MIC value (µM) single	FICI	RESULT
1	3i	25	50	0.50	Synergy
	Vancomycin	0.03	4		
2	3i	25	50	0.50	Synergy
	Vancomycin	0.01	4		
3	3i	25	50	0.50	Synergy
	Vancomycin	0.009	4		
4	3i	25	50	0.50	Synergy
	Vancomycin	0.004	4		
5	3i	12.5	50	0.32	Synergy
	Vancomycin	0.31	4		
6	3i	12.5	50	0.28	Synergy
	Vancomycin	0.15	4		
7	3i	12.5	50	0.26	Synergy
	Vancomycin	0.07	4		
8	3i	12.5	50	0.25	Synergy
	Vancomycin	0.03	4		
9	3i	12.5	50	0.25	Synergy
	Vancomycin	0.01	4		
10	3i	12.5	50	0.25	Synergy
	Vancomycin	0.009	4		
11	3i	12.5	50	0.25	Synergy
	Vancomycin	0.004	4		
12	3i	6.25	50	0.43	Synergy
	Vancomycin	1.25	4		
13	3i	6.25	50	0.28	Synergy
	Vancomycin	0.62	4		
14	3i	6.25	50	0.20	Synergy
	Vancomycin	0.31	4		
15	3i	6.25	50	0.16	Synergy
	Vancomycin	0.15	4		
16	3i	6.25	50	0.14	Synergy
	Vancomycin	0.07	4		
17	3i	6.25	50	0.13	Synergy
	Vancomycin	0.03	4		
18	3i	6.25	50	0.12	Synergy
	Vancomycin	0.01	4		
19	3i	6.25	50	0.12	Synergy
	Vancomycin	0.009	4		
20	3i	6.25	50	0.12	Synergy
	Vancomycin	0.004	4		
21	3i	3.12	50	0.37	Synergy
	Vancomycin	1.25	4		
22	3i	3.12	50	0.21	Synergy
	Vancomycin	0.62	4		

23	3i	3.12	50	0.14	Synergy
	Vancomycin	0.31	4		
24	3i	1.5	50	0.34	Synergy
	Vancomycin	1.25	4		
25	3i	1.5	50	0.18	Synergy
	Vancomycin	0.62	4		
26	3i	1.5	50	0.10	Synergy
	Vancomycin	0.31	4		
27	3i	0.7	50	0.32	Synergy
	Vancomycin	1.25	4		
28	3i	0.7	50	0.17	Synergy
	Vancomycin	0.62	4		
29	3i	0.7	50	0.09	Synergy
	Vancomycin	0.31	4		

TABLE 4: Indifference/Additive FIC index between standard antibiotic (Vancomycin) and compound (3i) against MRSA (Methicillin-Resistant *Staphylococcus aureus*)

S. No.	Compound	MIC value (μM) in combination	MIC value (μM) single	FICI	RESULT
1	3i	50	50	1.62	Indifference/ Additive
	Vancomycin	2.5	4		
2	3i	50	50	1.31	Indifference/ Additive
	Vancomycin	1.25	4		
3	3i	50	50	1.15	Indifference/ Additive
	Vancomycin	0.62	4		
4	3i	50	50	1.07	Indifference/ Additive
	Vancomycin	0.31	4		
5	3i	50	50	1.03	Indifference/ Additive
	Vancomycin	0.15	4		
6	3i	50	50	1.01	Indifference/ Additive
	Vancomycin	0.07	4		
7	3i	50	50	1.0	Indifference/ Additive
	Vancomycin	0.03	4		
8	3i	50	50	1.0	Indifference/ Additive
	Vancomycin	0.01	4		
9	3i	50	50	1.0	Indifference/ Additive
	Vancomycin	0.009	4		
10	3i	50	50	1.0	Indifference/ Additive
	Vancomycin	0.004	4		
11	3i	25	50	1.75	Indifference/ Additive
	Vancomycin	5	4		
12	3i	25	50	1.12	Indifference/ Additive
	Vancomycin	2.5	4		
13	3i	25	50	0.81	Indifference/ Additive
	Vancomycin	1.25	4		
	3i	25	50	0.65	Indifference/

14	Vancomycin	0.62	4		Additive
15	3i	25	50	0.57	Indifference/ Additive
	Vancomycin	0.31	4		
16	3i	25	50	0.53	Indifference/ Additive
	Vancomycin	0.15	4		
17	3i	25	50	0.51	Indifference/ Additive
	Vancomycin	0.07	4		
18	3i	12.5	50	1.5	Indifference/ Additive
	Vancomycin	5	4		
19	3i	12.5	50	0.87	Indifference/ Additive
	Vancomycin	2.5	4		
20	3i	12.5	50	0.56	Indifference/ Additive
	Vancomycin	1.25	4		
21	3i	12.5	50	1.00	Indifference/ Additive
	Vancomycin	0.03	4		
22	3i	6.25	50	1.37	Indifference/ Additive
	Vancomycin	5	4		
23	3i	6.25	50	0.75	Indifference/ Additive
	Vancomycin	2.5	4		
24	3i	3.1	50	1.31	Indifference/ Additive
	Vancomycin	5	4		
25	3i	3.1	50	0.68	Indifference/ Additive
	Vancomycin	2.5	4		
26	3i	1.5	50	1.28	Indifference/ Additive
	Vancomycin	5	4		
27	3i	1.5	50	0.65	Indifference/ Additive
	Vancomycin	2.5	4		
28	3i	0.7	50	1.26	Indifference/ Additive
	Vancomycin	5	4		
29	3i	0.7	50	0.63	Indifference/ additive
	Vancomycin	2.5	4		

8. References:

Sebbar. N. K., Ellouz, M., Essassi, E. M., Ouzidan, Y., & Mague, J. T. (2015). Crystal structure of 4-benzyl-2H-benzo [b][1, 4] thiazin-3 (4H)-one. *Acta Crystallographica Section E: Crystallographic Communications*, *71*(12), o999-o999.ISSN 2056-9890.

Dolomanov. O.V., Bourhis, L.J., Gildea. R.J., Howard, J.A.K., Puschmann, H. OLEX2: (2009). A Complete Structure Solution, Refinement and Analysis Program. Journal of Applied Crystallography, 42, 339-341.. http://dx.doi.org/10.1107/S0021889808042726

Swain. S. S., Paidesetty, S. K., & Padhy, R. N. (2019). Synthesis of novel thymol derivatives against MRSA and ESBL producing pathogenic bacteria. *Natural product research*,*33*(22), 3181-3189. https://doi.org/10.1080/14786419.2018.1474465.

Frisch, M. J. E. A gaussian 09, Revision d. 01, Gaussian. *Inc, Wallingford CT*. **2009**, *201*.

Sirianni, D. A., Alenaizan, A., Cheney, D. L., & Sherrill, C. D. (2018). Assessment of density functional methods for geometry optimization of bimolecular van der Waals complexes. *Journal of Chemical Theory and Computation*. *14*(6), 3004-3013. https://doi.org/10.1021/acs.jctc.8b00114.
